# O-GlcNAc of STING mediates antiviral innate immunity

**DOI:** 10.1186/s12964-024-01543-8

**Published:** 2024-03-01

**Authors:** Yujia Li, Wang An, Liyuan Lu, Jiali Yuan, Danhui Wu, Qi Yang, Jinrong Guo, Jingyu Yang, Mengjie Liu, Kaiyue He, Xinyuan Lei, Zhi-Xiang Xu

**Affiliations:** School of Life Sciences, Henan University, Kaifeng, 475004 Henan China

**Keywords:** STING, O-GlcNAcylation, Antiviral innate immunity, HSV-1, HBP

## Abstract

**Background:**

O-GlcNAcylation modification affects multiple physiological and pathophysiolocal functions of cells. Altered O-GlcNAcylation was reported to participate in antivirus response. Stimulator of interferon genes (STING) is an adaptor mediating DNA virus-induced innate immune response. Whether STING is able to be modified by O-GlcNAcylation and how O-GlcNAcylation affects STING-mediated anti-DNA virus response remain unknown.

**Methods:**

Metabolomics analysis was used for detecting metabolic alterations in HSV-1 infection cells. Succinylated wheat germ agglutinin (sWGA), co-immunoprecipitation, and pull-down assay were employed for determining O-GlcNAcylation. Mutagenesis PCR was applied for the generation of STING mutants. WT and *Sting1*^*−/−*^ C57BL/6 mice (KOCMP-72512-*Sting1*-B6NVA) were infected with HSV-1 and treated with O-GlcNAcylation inhibitor for validating the role of STING O-GlcNAcylation in antiviral response.

**Results:**

STING was functionally activated by O-GlcNAcylation in host cells challenged with HSV-1. We demonstrated that this signaling event was initiated by virus infection-enhanced hexosamine biosynthesis pathway (HBP). HSV-1 (or viral DNA mimics) promotes glucose metabolism of host cells with a marked increase in HBP, which provides donor glucosamine for O-GlcNAcylation. STING was O-GlcNAcylated on threonine 229, which led to lysine 63-linked ubiquitination of STING and activation of antiviral immune responses. Mutation of STING T229 to alanine abrogated STING activation and reduced HSV-1 stimulated production of interferon (IFN). Application of 6-diazo-5-oxonorleucine (DON), an agent that blocks the production of UDP-GlcNAc and inhibits O-GlcNAcylation, markedly attenuated the removal of HSV-1 in wild type C57BL/6 mice, leading to an increased viral retention, elevated infiltration of inflammatory cells, and worsened tissue damages to those displayed in STING gene knockout mice. Together, our data suggest that STING is O-GlcNAcylated in HSV-1, which is crucial for an effective antiviral innate immune response.

**Conclusion:**

HSV-1 infection activates the generation of UDP-Glc-NAc by upregulating the HBP metabolism. Elevated UDP-Glc-NAc promotes the O-GlcNAcylation of STING, which mediates the anti-viral function of STING. Targeting O-GlcNAcylation of STING could be a useful strategy for antiviral innate immunity.

**Supplementary Information:**

The online version contains supplementary material available at 10.1186/s12964-024-01543-8.

## Background

Host cells use various pattern recognition receptors (PRRS) to detect viral RNA and DNA to activate the production of type I interferons (T1IFNs) and inflammatory cytokines through a series of molecular responses to combat viral infection and coordinate innate and adaptive immunity. The immune response to pathogens is initiated upon detection of pathogen-associated molecular patterns (PAMPs) such as lipopolysaccharide, flagellin, and nucleic acids. Double-stranded DNA (dsDNA) is an important PAMP from multiple pathogens, including mycobacterium tuberculosis and herpes simplex virus-1 (HSV-1) [1–3].

T1IFNs are widely presumed to be the primary output of stimulator of interferon genes (STING) signaling during antiviral defense [4]. STING is a transmembrane protein on endoplasmic reticulum (ER), where it acts as a sensor for cytosolic second messenger, the cyclic dinucleotides (CDNs) [5, 6]. When cytoplasm DNAs, such as those from pathogens or damaged nuclear DNA, are detected by the cell, cyclic GMP-AMP synthase (cGAS) signal is activated. cGAMP binds to the V-shaped dimer pocket formed in the cytoplasmic region of STING, leading to the conformational changes of STING and translocation to the Golgi complex, where TBK1 is recruited and activated [7, 8].

STING is regulated by posttranslational modifications (PTMs). At least 3 different E3 ubiquitin ligases have been confirmed to play a positively regulatory role in STING activation. E3 ubiquitin ligases TRIM32 and TRIM56 promote K63-linked ubiquitination of STING, hence activating STING and its downstream signaling [9, 10]. The E3 ubiquitin ligase complex of ER composed of AMFR-GP78 and INSIG1 promoted K27-linked ubiquitination of STING, leading to the recruitment of TBK1 into STING complex and induction of interferon [11]. In contrast, the E3 ubiquitin ligases RNF5 and TRIM30α promote the K48-linked ubiquitination of STING, resulting in its degradation by the proteasome, ultimately inhibiting viral DNA or host cell DNA fragments-induced T1IFN production [12, 13]. Phosphorylation of human STING at Ser366 promotes the recruitment and activation of TBK1 and IRF3 [8]. The latter then enters the nucleus and activates transcription of IFN [14].

Zhang et al. reported that intracellular glucose metabolism is involved in the regulation of RIG-I-like receptors (RLRs) signaling and the induction of T1IFN, demonstrating the role of bioenergetics in the regulation of innate immunity [15]. Glucose metabolism is also closely related to the functional regulation of immune cells, such as macrophages [16] and T cells [17]. However, the detailed mechanism linking glucose metabolism to the innate antiviral defense remains to be elucidated.

Upon uptake through the glucose transporters, glucose fluxes into three major pathways, including glycolysis, the pentose phosphate pathway (PPP), and the hexosamine biosynthesis pathway (HBP) [18], whose metabolites not only work as substrates for the generation of bioenergetics, but also regulate biomass synthesis and gene expression of cells. 2–5% of intracellular glucose enters into the HBP to produce UDP-acetyl glucosamine (UDP-GlcNAc), which acts as a donor for glycosylation [19]. O-GlcNAcylation is considered to be a nutrient and stress sensor involved in transcription and translation, leading to the regulation of signal transduction and metabolic cellular processes [20]. O-GlcNAc transferase (OGT) mediates the transfer of UDP-GlcNAc to serine or threonine residues of target proteins and facilitates the O-GlcNAcylation modification [19, 21]. Previous studies have revealed essential roles of HBP and protein O-GlcNAcylation in the development of diabetes, cancer, and neurodegenerative diseases [19, 22–24].

O-GlcNAcylation is also associated with immune responses, including renewal of T-cell progenitors, Treg lineage instability, B-cell maturation, and macrophage polarization [25–28]. Several immune response-related transcriptional factors have been reported to be regulated by O-GlcNAcylation, including NF-kB, NFAT, and forkhead box P3 (FOXP3), fork head box O1 (FOXO1), MORC family CW-type zinc finger 2 (MORC2) and mitochondrial proteins, such as mitochondria antiviral signaling protein (MAVS), dynamin-related protein 1 (Drp1), and voltage-dependent anion channel (VDAC) [18, 25, 26, 29–33].

In the current study, we demonstrated that STING was O-GlcNAcylated on T229 by OGT and the PTM was enhanced by DNA virus infection. O-GlcNAcylation of STING enhanced its K63-linked ubiquitination to activate antiviral immune response. Blockade of O-GlcNAcylation rendered mice more susceptible to DNA virus infection, as did genetic deletion of STING in vivo. Together, our findings demonstrate a new mechanism whereby glucose metabolic reprogramming in HBP boosts antiviral immunity by promoting STING O-GlcNAcylation.

## Methods

### Mice and cells

WT and *Sting1*^*−/−*^ C57BL/6 mice (KOCMP-72512-*Sting1*-B6NVA) were purchased from Cyagen Biosciences Inc. (Suzhou, China). All animals were housed under specific pathogen–free conditions at 21 °C and 31% humidity. The mice were weaned at 28 days after birth and identified in separate cages. Food and water were provided ad libitum, and health checks were performed daily. The padding was changed weekly. The treatment of the mice followed the guidelines set by the Henan University Medical Animal Center, the National Institute of Health Laboratory Animal Care and Use Guidelines, and the school Animal Care and Use Committee. All mouse studies were approved by the Medical Ethics and Laboratory Animal Welfare Committee of Henan University School of Medicine. Adult female and male mice were raised in the same cage and allowed to mate freely. Mouse embryonic fibroblasts (MEFs) were isolated from WT and *Sting1*^−/−^ mouse embryos. MEFs were cultured with DMEM supplemented with 10% fetal bovine serum (FBS). HEK-293 T cells were purchased from ATCC. Cells were grown in RPMI-1640 medium (Corning Life Sciences) supplemented with 10% FBS, 100 U/mL penicillin, and 100 μg/mL streptomycin. Cells were cultured at 37℃ with 5% CO2.

### Generation of shRNA-STING, human STING-Flag, and STING mutant cell line

To generate the full length of STING with mutations, lenti-CRISPR v3 vector containing FLAG-tagged and HA-tagged full-length STING was used as a template for PCR. All primers used for cloning were listed in Additional file 1: Table S1. A series of STING mutants targeting its potential O-GlcNAcylation sites were constructed. STING S4A/S5A, S5A, S195A, T229A, S305A, S322A, T354A, T348A/S349A, T376A/S379A, T354A, and S379A were constructed. To generate STING mutant constructs, the upstream and downstream primers of mutant fragments were constructed for amplification. Primers used for mutagenesis PCR of STING were listed in Additional file 1: Table S1. The complete nucleotide sequences of STING mutants were confirmed by sequencing.

### Generation of cell line stably expressing STING

KYSE-30 and HEK-293 T with STING knockdown or STING-WT and STING-T229A overexpression, and *Sting1*^*−/−*^ MEFs with STING-WT and STING-T229A overexpression were routinely cultured in RPMI-1640 medium (Cat # 10,040-CVR, Corning) supplemented with 10% FBS (Cat # 04–001-1Acs, BI). The specific lenti-CRISPR v3 plasmid, lentivirus packaging plasmid psPAX2, and envelope plasmid pMD2.G were co-transfected into 293 T cells in 100 mm culture dishes using Lipofectamine 2000 (Cat # 11,668,019, Invitrogen). Forty-eight hours after the transfection, cell culture supernatant was harvested and centrifuged at 2,000 rpm for 10 min and then filtered through a 0.45 μm aperture (Cat # IPVH00010, Millipore) to remove cells. When recipient cells were grown to ∼70% confluence, media containing specific lenti-CRISPR v3 lentivirus and polybrene (8 μg/ml) (Cat # H8761, Solarbio) were applied to the cells. After selection with puromycin (Cat # P8230, Solarbio), single colonies of the cells were picked up for enlarged culture. Cells were confirmed by quantitative RT-PCR and Western blotting. Cells were maintained under a humidified atmosphere of 5% CO2 at 37℃ in RPMI 1640 supplemented with 10% FBS.

### Culture of MEFs

Adult female and male mice were raised in the same cage and allowed to mate freely. The time when vaginal plugs were detected was recorded as 0.5 days of pregnancy (0.5 dpc). The mice were sacrificed at 13.5 days of pregnancy. The abdominal cavity was opened, and the fat and mesangium were trimmed with iris scissors and tweezers. The beaded uterus was removed and placed in the first 100 mm dish. The uterine wall was opened and the embryos were removed one by one and placed in a 60 mm dish. The head, limbs, tail and viscera of mice were removed, and the remaining tissues were placed in a 35 mm dish and cut to the size of 1 mm^3^. The cut tissue was sucked into a 15 mL centrifuge tube and centrifuged at 1,000 rpm at room temperature for 3—5 min to remove the supernatant. MEFs were cultured in DMEM supplemented with 10% FBS.

### qPCR

Total RNAs were extracted and reverse-transcribed into cDNA. qPCR was performed using ChamQ Universal SYBR qPCR Master Mix from Vazyme company (Cat # Q711-02). The fold change in mRNA expression was determined by a standard 2^^−ΔΔct^ method. GAPDH was analyzed as an internal control. All experiments were repeated at least 3 times. A series of upstream and downstream primers of target molecules were designed for PCR amplification. Primer sequences of qRT-PCR were provided in Additional file 1: Table S1.

### Immunoblotting

Proteins with different molecular weights were separated by denatured SDS-PAGE gel and then transferred to PVDF membrane (Cat # IPVH00010, Millipore), which was blocked with nonfat milk. After the target protein was fully combined with the antibody, the excess unbound primary antibody was eluted with TBST, and the corresponding HRP labeled secondary antibody was added. When the chemiluminescent solution was applied, target protein was developed by using chemiluminescence instrument. Antibodies used in Western blotting are shown in Additional file 2: Table S2.

### Immunohistochemistry

Tissue samples were fixed in 4% paraformaldehyde and embedded in paraffin according to standard procedures [34]. IHC staining is performed according to the manufacturer’s protocol (Cat # C516337, Sangon Biotechnologies, Shanghai, China).

### Co-immunoprecipitation

Cells were collected and lysed using lysis buffer [50 mM Tris–HCl (pH 7.4), 150 mM NaCl, 1% NP-40, 0.1% SDS]. Lysates were incubated with specific antibody at 4 °C for overnight. The following day, protein A/G agarose (Cat # 37,478, Cell signaling technology) beads were added and incubated at 4 °C for 2 h. Beads were washed 5 times with washing buffer, and bound proteins were separated using SDS-PAGE followed by subsequent immunoblotting analysis.

### Succinylated wheat germ agglutinin (sWGA) pull-down assay

Cells were lysed in RIPA buffer (Cat # P0013B, Beyotime Biotechnology, Shanghai, China) containing 50 mM Tris–HCl (pH 7.4), 150 mM NaCl, 1% NP-40, 0.1% SDS. Succinylated wheat germ agglutinin (sWGA)-conjugated agarose beads (Cat # AL-1023S-2, Vector Laboratories) were cleaned in washing buffer containing 1/3 RIPA buffer (Cat # P0013B, Beyotime) and 2/3 PBS plus PhosSTOP (Cat # 04906845001, Roche) and EDTA-free protease inhibitors (Cat # 04693159001, Roche). Pre-cleaned lysates were incubated overnight with sWGA-conjugated agarose beads in 4℃. Precipitated complexes were eluted with washing buffer. Loading buffer (5X) (Cat # P1040, Solarbio) and precipitated complexes were mixed and denatured at 100 °C for 5 min. Precipitated complexes were immunoblotted with the indicated antibodies.

### ELISA and measurement of UDP GlcNAc

Murine blood was collected and left for half an hour before centrifugation at 2,000 rpm for 1 min for isolating the plasma. Cytokines in the plasma and supernatant of cell culture were quantified using ELISA kits for human IL-6 (Cat # YJ028583, mlbio) and IFN-β (Cat # YJ710284, mlbio) or mouse IL-6 (Cat # YJ063159, mlbio) and IFN-β (Cat # YJ720131, mlbio) according to the manufacturer’s protocol.

For the detection of UDP-GlcNAc, an ELISA kit from Mlbio LLC (Cat # JL45954) was used. The measurement was performed following the manufacturer’s instruction.

### Metabolomics analysis

Cell lysates were collected and vortexed for 10 s. 50 μL of the sample was transferred to a centrifuge tube, mixed with 250 μL 20% acetonitrile/methanol, vortexed for 3 min, and centrifuged at 4℃ for 10 min at 12,000 r/min. 250 μL of the supernatant was transferred into a new centrifuge tube and stood at -20 ℃ for 30 min, followed by centrifuging at 12,000 r/min for 10 min at 4℃. After centrifugation, 180 μL of the supernatant was transferred into a protein precipitating plate for LC–MS analysis.

### In vitro viral infection and titer assay

The herpes simplex virus 1 (HSV-1) (Cat # HSV-H129-G4) was purchased from Shanghai Gene Company, and kept in -80 ℃. KYSE-30 cells and MEFs were infected with HSV-1 (KOS strain; MOI = 1) for 16 h. Viral gD gene in cells was qualified by qPCR. Experiments involving in HSV-1 were performed in strict accordance with laboratory biosafety standards of Henan University.

### HSV-1 challenge in mice

Mice were infected with HSV-1 (Cat # H129-G4) from Shanghai Gene Company by intravenous (i.v.) injection, WT and *Sting1*^−/−^ mice (7—10 weeks old) were infected with HSV-1 (5 × 10^6^ PFU/mouse for immunological studies), as previously described [35]. Serum and tissues including spleen, liver, and lung were collected at 6 days post injection for immunological and histological analyses. Mice were treated with 1 mg/kg of oxo-L-norleucine (DON) (Cat # HY-108357, MCE) dissolved in 200 μl PBS by i.p. on day 1 to day 3, and then administered every other day until the end of the experiment.

### Statistical analysis

All experiments were performed with a minimum of three independent replications. Data were presented as mean ± SEM. All data meet the assumptions of the *t*-ests. Student’s unpaired t-test was used to compare the means of two groups. One-way analysis of variance (ANOVA) was used to compare three or more groups. Statistical significance was represented as: not significant (NS), *p* > 0.05; *, *p* ≤ 0.05; **, *p* ≤ 0.01; ***, *p* ≤ 0.001; and ****, *p* ≤ 0.0001.

## Results

### HBP is actively enhanced in HSV-1 infected mice

**Fig. 1:**
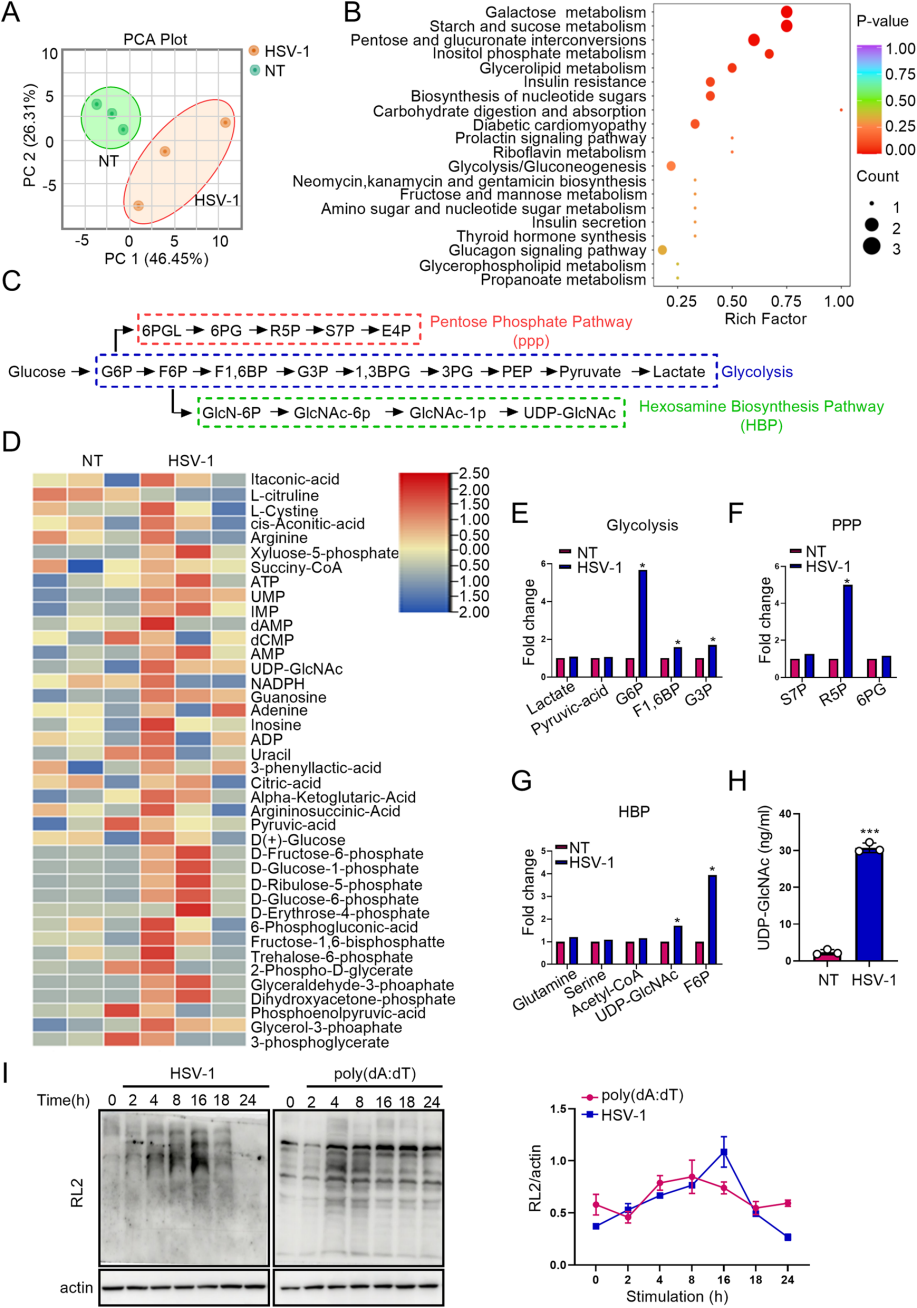
HSV-1 Infection Activates Glucose Metabolism. A and B C57BL/6 mice (6 weeks old) were challenged with HSV-1 (5 × 10.^6^ PFU/mouse) for 6 days. The spleens of mice were collected for a targeted metabolomics assay. Glucose metabolites determined by LC–MS/MS metabolomics were assessed by principle-component analysis (**A**) and pathway-enrichment analysis (**B**). **C** Metabolites in the glycolysis, PPP, and HBP. **D**–**G** Heatmap of glucose metabolites (**D**) and fold changes of intermediate metabolites in the glycolysis (**E**), PPP (**F**), and HBP (**G**) in HSV-1-treated mice. **H** Levels of UDP-GlcNAc in the serum of non-treated and HSV-1-challenged mice were determined with an UDP-GlcNAc kit. **I** O-GlcNAc in KYSE-30 cells transfected with poly(dA:dT) (2 μg/mL) or treated with HSV-1 (MOI = 1) was detected with immunoblotting. Quantification of immunoblots was performed with ImageJ. Data are representatives of 3 independent biological replicates. Data are means ± SEM. * *p* < 0.05, *** *p* < 0.0001

HSV-1 is a neurotropic DNA virus that is transmitted via mucosal tissues (typically oral, ocular, or genital) and infects epithelial cells before reaching the central nervous system where it establishes latency in neurons [36, 37]. Spleens of *Sting1*-deficient mice injected with HSV-1 intravenously (i.v.) possesse higher titers of HSV-1 as compared with those in spleens of wild-type mice [38]. An integrated STING signaling results in a greater antiviral activity [38]. Li et al. [18] found that compared with wild type mice, *Ogt*-deficient mice contain significantly higher levels of VSV transcript in the spleen, liver, and lung, indicating insufficient control of viral replication when O-Glycosylation is suppressed. HBP, a branch of glucose metabolism, provides UDP-GlcNAc for O-Glycosylation. Thus, we postulated that glucose metabolism may affect antiviral response through providing donor UDP-GlcNAc for O-Glycosylation. To achieve a comprehensive understanding of metabolic changes in response to viral infection, we performed a targeted metabolomics assay in mice challenged with HSV-1. Principal-component analysis revealed a markedly altered metabolic profile in the spleens of mice exposed to HSV-1 (Fig. 1A). Pathway enrichment analysis identified several crucial metabolic pathways were altered; they were primarily involved in galactose, starch and sucrose, pentose and glucoronate interconversions, inositol phosphate, glycerolipid and insulin resistance metabolism (Fig. 1B, C). Metabolites involved in two major glucose metabolic pathways, glycolysis and the pentose phosphate pathway (PPP), were increased upon HSV-1 challenge (Fig. 1D-F). These findings were consistent with previous studies showing that viral infections activate aerobic glycolysis and PPP [39, 40] and that glucose utilization plays an essential role in promoting antiviral defense response to influenza infection [41]. Fructose-6 phosphate (F6P), a metabolite being shunted from the glycolysis to HBP, was elevated upon HSV-1 challenge (Fig. 1G). Physiologically, 2–3% of F6P enters into the HBP to produce UDP-GlcNAc in the presence of glutamine, acetyl-CoA, and UTP. This finding was further supported by an elevated activation of HBP and increased production of UDP-GlcNAc in the serum of mice treated with HSV-1 when compared with that in control mice (Fig. 1H).

UDP-GlcNAc acts as a donor for glycosylation modifications. Thus, we tested O-GlcNAcylation in HSV-1-infected (MOI = 1) KYSE-30 cells, a cell line generated from human esophageal squamous cell carcinoma (ESCC). Levels of O-GlcNAcylated proteins were substantially increased (Fig. 1I). We also challenged KYSE-30 cells with poly(dA:dT), a poly (deoxyadenylic-deoxythymidylic) acid sodium salt. Poly(dA:dT) forms a double-stranded DNA of poly(dA-dT):poly(dT-dA), a synthetic analog of B form DNA (B-DNA), the canonical right-handed DNA helix. Consistent with the finding in DNA virus (HSV-1)-infected cells, transduction of poly(dA:dT) similarly led to a striking elevation of O-GlcNAcylation in KYSE-30 cells (Fig. 1I). Collectively, our data suggest that HSV-1 (viral DNA mimics) promotes glucose metabolism of host cells with a marked increase in HBP, which provides donor sugar for O-GlcNAcylation.

### O-GlcNAcylation promotes antiviral immune response

**Fig. 2:**
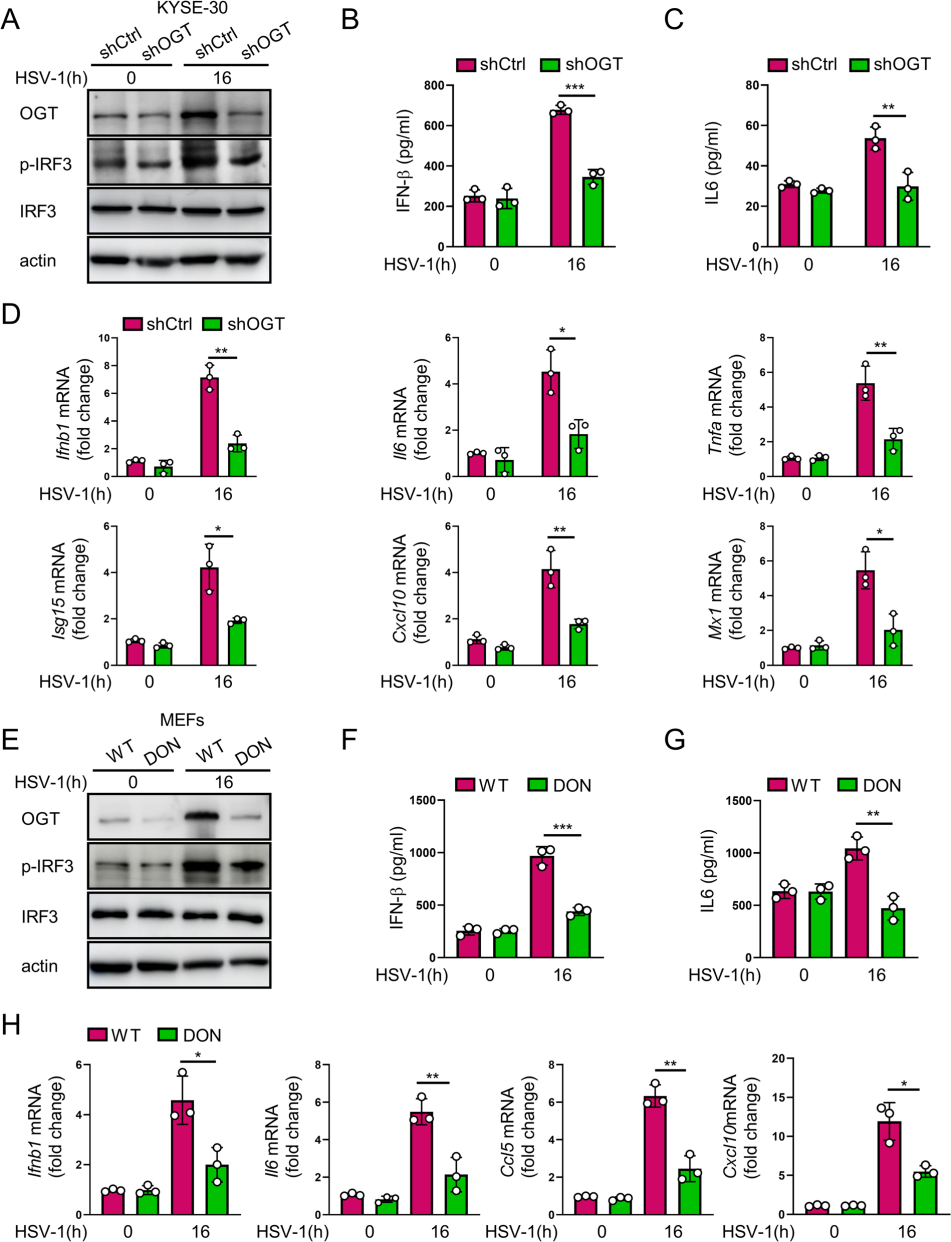
O-GlcNAcylation promotes antiviral immune response. **A** Immunoblotting of phosphorylated IRF3 (p-IRF3), IRF3, OGT, and actin in shCtrl and shOGT cells infected with HSV-1 (MOI = 1) for 16 h. **B** and **C** Levels of IFN-β and IL-6 in the supernatants from shCtrl and shOGT cells challenged with HSV-1 (MOI = 1) for 16 h. **D** qRT-PCR analysis of *Ifnb1*, *Il6*, *Tnfa*, *Isg15*, *Cxcl10*, and *Mx1* mRNA level in KYSE-30 cells treated as in (**A**). **E** Immunoblotting of phosphorylated IRF3 (p-IRF3), IRF3, OGT, and actin in MEFs infected with HSV-1 (MOI = 1) for 16 h with or without cotreatment of DON (10 μM) for 12 h. **F** and **G** Levels of IFN-β and IL-6 in the supernatants from MEFs treated as in (**E**). **H** qRT-PCR analysis of *Ifnb1*, *Il6*, *Ccl5*, and *Cxcl10* mRNA in KYSE-30 cells treated as in (**E**). Data represent mean ± SEM. * *P* < 0.05, ** *P* < 0.01, *** *P* < 0.001, compared with shR-OGT or DON treatment, *n* = 3

To determine whether O-GlcNAcylation is involved in the host antiviral immunity, we constructed an OGT-knocked down KYSE-30 cell line. Both control and OGT-depleted cells were challenged with HSV-1. We found that OGT-deficient cells exhibited a marked reduction in IRF3 activation (Fig. 2A), as well as a decrease in the level of IFN-β and IL-6 as compared with those in control KYSE-30 cells upon the HSV-1 challenge (Fig. 2B, c). Consistently, knockdown of OGT led to a marked decrease in the transcription of IFN-stimulated genes (ISGs), such as *Ifnb1*, *Il6*, *Tnfa*, *Isg15*, *Cxcl10*, and *Mx1,* in HSV-1-treated KYSE-30 cells (Fig. 2D). To further determine the role of O-GlcNAcylation in antiviral immune response, we treated MEFs with HSV-1 in the presence or absence of DON, a glutamine antagonist and an inhibitor of O-GlcNAcylation [42, 43]. It showed that inhibition of HBP (O-GlcNAcylation) markedly reduced HSV-1-induced IRF3 phosphorylation and T1IFN signal-related cytokine release (Fig. 2E-H). In addition, we challenged control and OGT-depleted cells with ISD and poly (dA:dT). As shown in HSV-1 infection cells, an upregulations of OGT was observed in cells challenged with ISD or poly (dA:dT). Depletion of OGT led to a marked reduction in IRF3 activation and poly(dA:dT)/ISD challenge-induced upregulation of OGT (Additional file 3: Fig. S1A, B). These data indicate that O-GlcNAcylation positively regulates host antiviral immune response against HSV-1.

### STING is O-GlcNAcylated

**Fig. 3:**
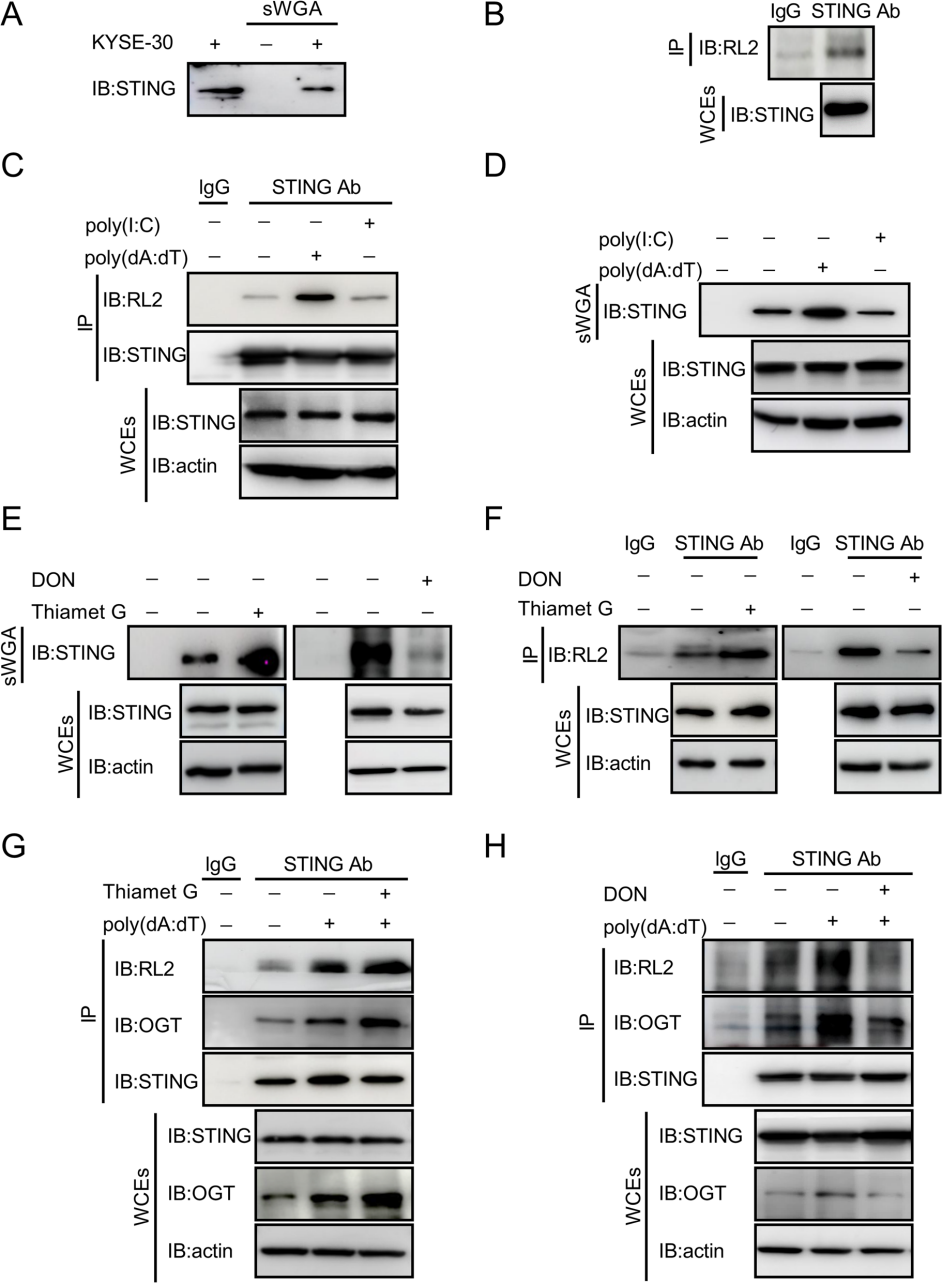
STING is O-GlcNAcylated. **A** O-GlcNAcylated proteins in KYSE-30 cells were pulled down with sWGA beads. STING was detected with an anti-STING antibody from abcam. **B** Immunoprecipitated STING was assessed for O-GlcNAcylation with a specific anti-O-GlcNAc antibody, RL2. **C** KYSE-30 cells were transduced with poly(dA:dT) (2 μg/mL) or poly(I:C) (4 μg/mL) for 16 h. STING in whole cell extracts (WCEs) was immunoprecipiated with an anti-STING antibody from abcam. O-GlcNAcylated STING was detected with an anti-O-GlcNAc monoclonal antibody, RL2. **D** WCEs of KYSE-30 cells pretreated with poly(dA:dT) (2 μg/mL) or poly(I:C) (4 μg/mL) for 16 h were isolated. O-GlcNAcylated proteins were pulled down with sWGA beads. STING in the pull-down complexes was detected with immunoblotting. WCEs were also used for the detection of STING with immunoblotting. Actin serves as a loading control. **E** O-GlcNAcylated proteins in KYSE-30 cells treated with TMG (10 μM) or DON (10 μM) for 12 h were pulled down with sWGA beads. STING in the pull-down complexes was detected with immunoblotting. WCEs were also analyzed with immunoblotting for the expression of STING and actin. Actin serves as a loading control. **F** STING in WCEs from KYSE-30 cells treated with TMG (10 μM) or DON (10 μM) for 12 h was immunoprecipiated with an anti-STING antibody. The O-GlcNAcylated STING was detected with an anti-O-GlcNAc monoclonal antibody, RL2. WCEs were also analyzed with immunoblotting for the expression of STING and actin. Actin serves as a loading control. **G** and **H** KYSE-30 cells were treated with TMG (**G**) or DON (**H**) and poly(dA:dT) (2 μg/mL) for 16 h. STING was immunoprecipitated with anti-STING antibody from abcam. RL2, OGT, and STING in the complex and in the input were detected with immunoblotting. Data are representatives from 3 independent experiments

To determine whether cGAS (DNA-sensor cyclic-GMP-AMP (cGAMP) synthase) is O-GlcNAcylated, we used sWGA to pull down O-GlcNAcylated proteins and then applied a cGAS antibody to detect O-GlcNAcylation of endogenous cGAS in KYSE-30 cells. It showed that cGAS was hardly to be pulled down by sWGA in our experimental setting (Additional file 3: Fig. S2A). STING is an adaptor conveying signals from the recognition of DNA viruses to the downstream responsive targets. To determine whether STING is O-GlcNAcylated, we used sWGA to pull down O-GlcNAcylated proteins and then applied a STING antibody to detect O-GlcNAcylation of endogenous STING in KYSE-30 cells. It showed that STING was pulled down by sWGA (Fig. 3A). Parallelly, we used STING antibody for a co-immunoprecipitation (co-IP) assay to pull down STING and detected with a pan-O-GlcNAcylation antibody RL2. STING was again detected to be O-GlcNAcylated (Fig. 3B). We then transfected the cells with poly(dA:dT), which mimics double-stranded viral DNA. Relative to untransfected cells, cells transduced with poly(dA:dT) displayed a marked increase in STING O-GlcNAcylation (Fig. 3C, D). Interestingly, STING O-GlcNAcylation was not altered in cells challenged with poly(I:C), a double strand RNA mimicking viral RNA (Fig. 3C, D). To further validate the O-GlcNAcylation of STING, we pretreated the cells with thiamet-G (TMG), an inhibitor of O-GlcNAcylase (OGA) and inducer of O-GlcNAcylation [44, 45]. TMG markedly promoted STING O-GlcNAcylation (Fig. 3E). In contrast, exposure to DON, a glutamine antagonist and hence suppression of O-GlcNAcylation [42, 43], blunted O-GlcNAcylation of STING, as compared with that in vehicle-treated cells (Fig. 3E, F).

OGT, which transfers UDP-GlcNAc to the serine or threonine residues of target proteins, is the only enzyme catalyzing O-GlcNAcylation. Cells stimulated with poly(dA:dT) bore an increased interaction between STING and OGT and O-GlcNAcylation of STING was also increased substantially, which was further enhanced by TMG treatment of the cells (Fig. 3G). In contrast, the level of STING O-Glycosylation was markedly reduced in cells treated with DON regardless of the presence of poly(dA:dT) and the interaction between STING and OGT was weakened (Fig. 3H). Taken together, these data demonstrate that STING is O-GlcNAcylated, which is enhanced by the viral DNA mimics.

### STING is O-GlcNAcylated on T229

**Fig. 4:**
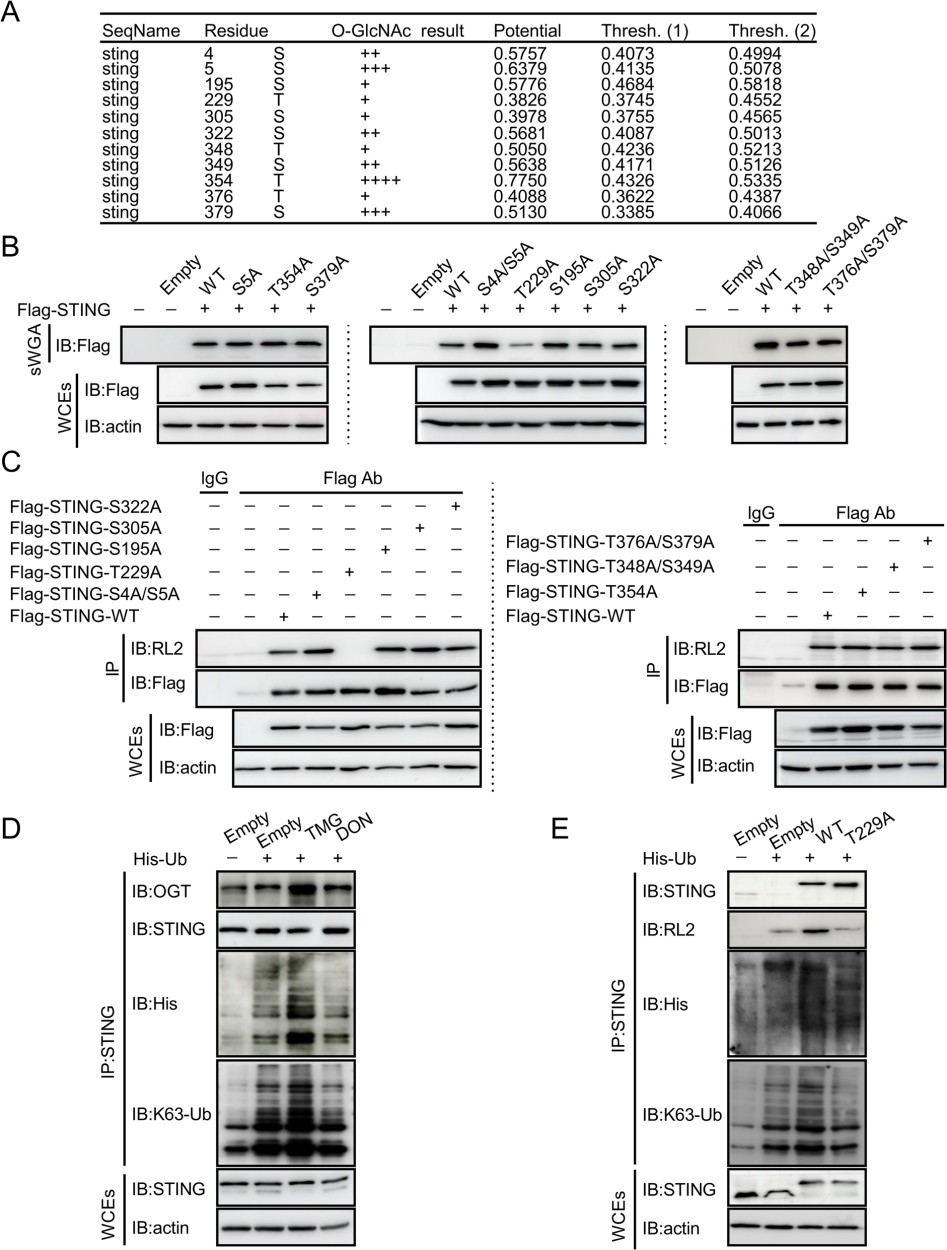
O-GlcNAcylation of STING on T229 promotes its K63-linked Ubiquitination. **A** O-GlcNAcylation residues in STING were analyzed and predicted with the Yin-O-Yang website. **B** and **C** A series of mutant constructs of STING were generated with mutations in individual residue predicted by the Yin-O-Yang website. STING in the pull-down complexes and in the WCEs was detected with immunoblotting (**B**). (**C**) STING and its mutants were immunoprecipitated with anti-Flag antibody. O-GlcNAcylated Flag-STING and its mutants were detected with an anti-O-GlcNAc monoclonal antibody, RL2. STING and its mutants in WCEs were detected with immunoblotting. **D** Immunoblotting of K63 ubiquitination in immunoprecipitated complex pulled down with STING antibody from lysates of cells treated with or without DON (10 μM) or TMG (10 μM) for 12 h. **E** HEK-293 T cells were transfected with FLAG-tagged STING-WT or -T229A in the presence of His-tagged ubiquitin. Co-IP was performed with an anti-STING antibody from abcam. Immunoblotting was performed with antibodies against His-tag (His-Ub), K63-Ub, RL2, and STING. Data are representatives from 3 independent experiments

To determine the O-GlcNAcylation site(s) on STING, we analyzed the amino acid sequence of STING with the Yin-O-Yang website (/Services/YinOYang/) and identified potential residues for the modification (Fig. 4A). We then constructed FLAG-tagged WT STING and its mutants (S5A, S4/S5A, S195A, T229A, S305A, S322A, T354A, T348A/S349A, and T376A/S379A). An initial screening of single and double mutations in transfected KYSE-30 cells was performed by using sWGA to pull down O-GlcNAcylated proteins followed by immunoblot with Flag antibody. It revealed that mutation at threonine 229 (T229A) led to a marked decrease in the O-GlcNAc of STING (Fig. 4B). We then used the Flag antibody to pull down ectopic Flag-STING, and detected with pan-O-GlcNAcylation antibody RL2. It confirmed that T229A mutation abrogated the O-GlcNAc of STING (Fig. 4C). Together, these results support our notion that STING is O-GlcNAcylated on T229.

### O-GlcNAcylation of STING promotes its K63-linked Ubiquitination

Both the K63 ubiquitination and S365 phosphorylation of STING have been reported as important regulators for STING-IRF3 interaction [46]. Thus, we asked whether O-GlcNAcylation of STING may possess a broad impact on the function of STING by regulating the ubiquitilation of the protein. We transduced KYSE-30 cells with STING constructs and detected the ubiquitination of STING in cells with O-GlcNAcylation manipulations. Treatment of TMG, an inhibitor of O-GlcNAcylase (OGA) and inducer of O-GlcNAcylation, increased K63 ubiquitination of STING, whereas exposure to DON, the HBP inhibitor, reduced total and K63-linked ubiquitination of STING (Fig. 4D). Further, we examined K63-linked ubiquitination of STING in the absence of OGT or after the treatment of DON. The level of K63-linked ubiquitination of STING decreased after OGT depletion or DON treatment (Additional file 3: Fig. S2B, C). Next, we co-transfected HEK-293 T cells with plasmids encoding polyhistidine (His)-tagged ubiquitin and WT and T229A STING. Ubiquitination of endogenous STING was readily detected, in particular in cells transduced with His-Ub (Fig. 4E, left two lanes). Consistently, co-transduction with His-Ub led to the ubiquitination of overexpressed WT STING (Fig. 4E, lane 3). However, STING T229A showed an attenuated K63-linked ubiquitination as compared with WT STING (Fig. 4E, lane 4).

In addition to K63-linked ubiquitination that promotes the activation of the STING pathway, K6-, K11-, and K27-linked ubiquitination also plays a role in STING activation and/or stabilization [11, 47–49]. For example, the K27-linked polyubiquitination of STING mediated by AMFR and INSIG1 facilitates TBK1 recruitment and activation [11]. The K6-linked polyubiquitination of STING mediated by RNF144A enhances STING translocation and promotes the activation of downstream signaling pathways [47]. Furthermore, RNF26 promotes the K11-linked polyubiquitination of STING and increases the stability of STING following viral infection [48, 49]. We investigated whether the O-GlcNAc of STING potentially enhances other types of ubiquitination on STING. We detected K27-linked ubiquitination of STING and found that there was no difference in K27-linked ubiquitination in STING T229A as compared with that in WT STING (Additional file 3: Fig. S2D). Several E3 ubiquitin ligases have been reported to play a positively regulatory role in STING activation. For example, E3 ubiquitin ligases TRIM32 and TRIM56 promote K63-linked ubiquitination of STING, hence activating STING and its downstream signaling [9, 10]. We explored whether increased ubiquitination induced by STING O-GlcNAc enhanced recruitment of TRIM56. STING T229A showed an attenuated K63-linked ubiquitination and recruitment of TRIM56 as compared with WT STING (Additional file 3: Fig. S2D). Together, these results indicate that the O-GlcNAc of STING enhances TRIM56 recruitment, increases K63-mediated ubiquitination of STING, and promotes the activation of STING (Additional file 3: Fig. S2D).

### O-GlcNAcylation of STING activates antiviral response

**Fig. 5:**
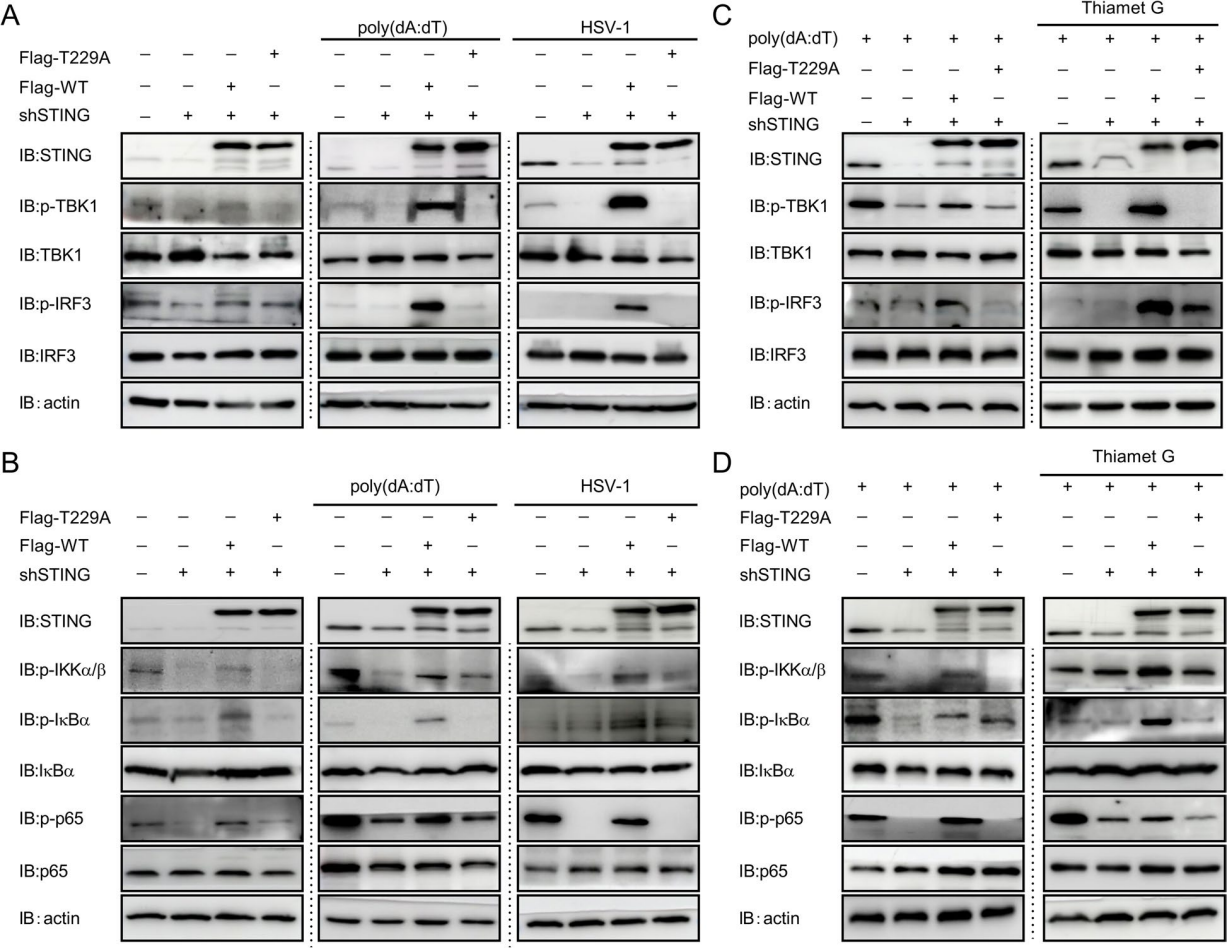
O-GlcNAcylation of STING activates antiviral signaling. **A** and **B** Downstream targets in STING signaling were detected with immunoblotting in shSTING cells reconstituted with either STING-WT or STING-T229A followed by challenges with HSV-1 (MOI = 1) or poly(dA:dT) (2 μg/mL) for 16 h. **C** and **D** Downstream targets in STING signaling in STING-WT- or STING-T229A-reconstituted shSTING cells challenged with poly(dA:dT) in the presence or absence of TMG (10 μM, O-GlcNAc activator) for 12 h. Data are representatives from 3 independent experiments

Increased HBP activity and STING O-GlcNAcylation upon HSV-1 challenge prompted us to hypothesize that STING O-GlcNAcylation may play a role in antiviral immune responses. STING is a transmembrane (TM) dimeric protein located on the endoplasmic reticulum (ER) or Golgi. STING is activated by binding of its cytoplasmic ligand-binding domain (LBD) to cyclic dinucleotides produced by the DNA-sensor cyclic-GMP-AMP (cGAMP) synthase (cGAS) or invading pathogens [50–52]. Cyclic dinucleotides induce a conformational change in the STING LBD, leading to high-order oligomerization of STING that is essential for triggering the downstream signaling pathways [7, 8, 36]. To determine the impact of O-GlcNAcylation modification on STING dimer formation and/or oligomerization, we performed a non-denaturing gel electrophoresis. The results of non-denaturing gel electrophoresis showed that O-GlcNAcylation modification contributed to the dimer formation and oligomerization of STING, which was absence in STING T229A (Additional file 3: Fig. S3A). STING activation requires trafficking from the ER to ER-Golgi intermediate compartment (ERGIC) and the Golgi, which is essential for the recruitment and activation of kinase TBK1 and transcription factor IRF3 that induces IFN expression [53, 54]. Immunofluorescence assay further revealed that DNA stimulation led to rapid STING trafficking from the ER to post-Golgi vesicles in WT STING cells (Additional file 3: Fig. S3B). However, this process was blocked in T229A STING cells (Additional file 3: Fig. S3B). Activation and transduction of canonical antiviral immune signaling, e.g. the TBK1-IRF3 and NF-kB pathways, by STING, are well defined to promote the production of inflammatory mediators in response to viral infection [55]. To test if STING O-GlcNAcylation affects its downstream antiviral response, we reconstituted shSTING KYSE-30 cells with either STING WT or T229A mutant through a lentivector-based transduction strategy. Knockdown of STING led to a decreased phosphorylation of TBK1, IRF3, IKKα/β, IkBα and p65 in the presence of HSV-1 or poly(dA:dT) challenge as compared with that in control KYSE-30 cells under similar treatment (Fig. 5A, B), supporting STING as a key mediator in antiviral response [56]. Reconstitution of STING-knocked down cells with WT STING fully rescued STING expression (Fig. 5A) and restored the activation of TBK1-IRF3 and NF-kB signaling upon HSV-1 or poly(dA:dT) challenge. However, STING-depleted cells reconstituted with STING T229A mutant failed to reactivate the TBK1-IRF3 and NF-kB signaling regardless of HSV-1 or poly(dA:dT) challenge (Fig. 5A, B). In addition, treatment of TMG failed to induce any increase in IRF3 and p65 phosphorylation in shSTING KYSE-30 cells reconstituted with STING T229A mutant as observed in WT STING-reconstituted cells (Fig. 5C, D). These results indicate that STING O-GlcNAcylation is necessary and sufficient for the activation of antiviral signaling.

### Depletion of STING O-GlcNAcylation abrogates IFN signaling

**Fig. 6:**
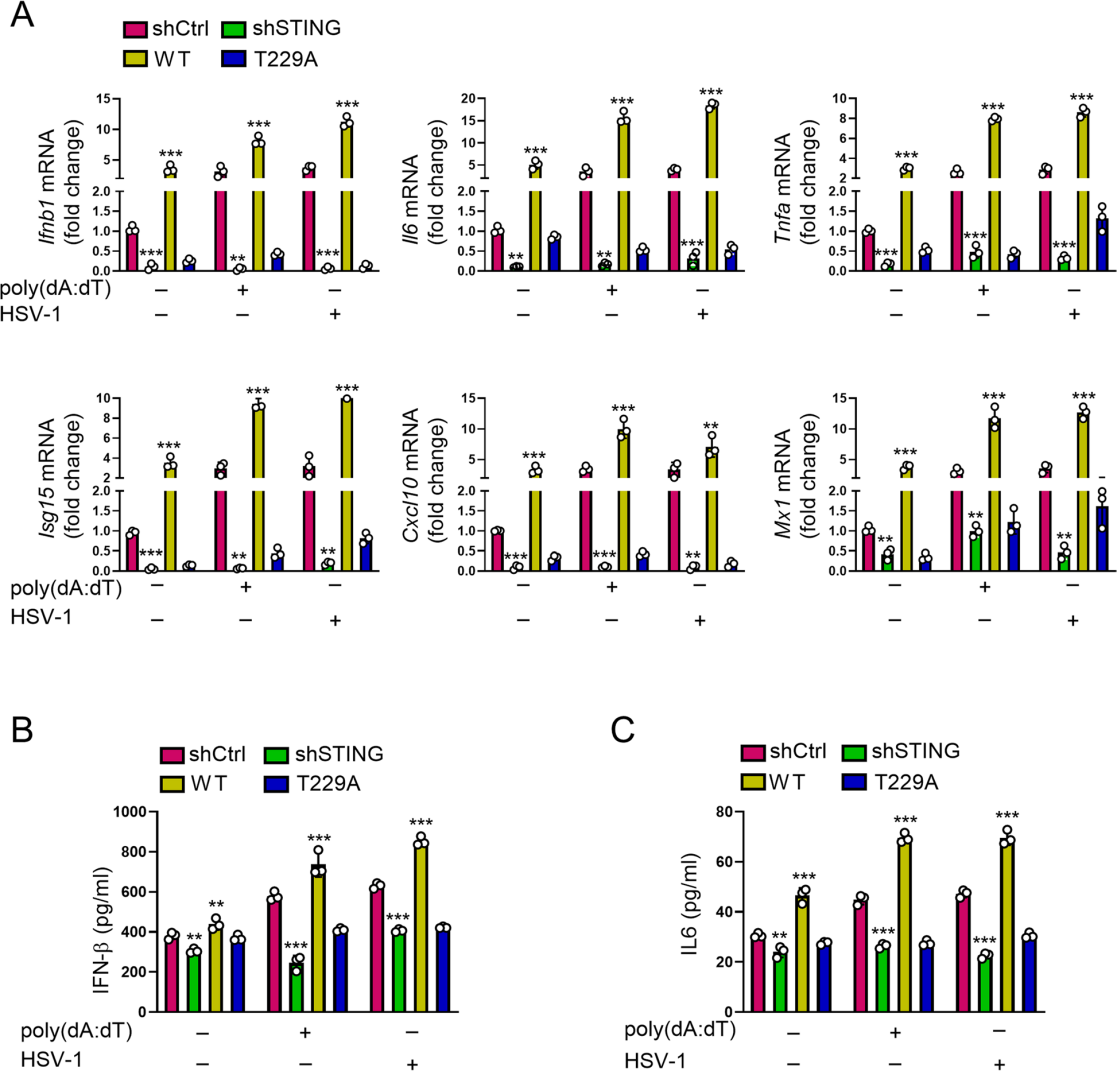
Sting-T229A mutation abrogates IFN signaling. **A** shSTING KYSE-30 cells reconstituted with either STING-WT or STING-T229A were stimulated with HSV-1 (MOI = 1) or transfected with 2 μg/mL poly(dA:dT) by Lipofectamine 2000 for 16 h. Levels of *Ifnb1*, *Il6*, *Tnfa*, *Isg15*, *Cxcl10*, and *Mx1* mRNA in cells were measured with RT-PCR. **B** and **C** shSTING KYSE-30 cells reconstituted with either STING-WT or STING-T229A were stimulated with HSV-1 (MOI = 1) or transfected with 2 μg/mL poly(dA:dT) by Lipofectamine 2000 for 16 h. Levels of IFN-β and IL-6 in the supernatants of the cell culture were measured with ELISA. Data are representatives of 3 independent biological replicates. Data are means ± SEM. ** *p* < 0.001. *** *p* < 0.0001

Activation of STING signaling leads to the generation of IFN and hence triggers antiviral immune response. To determine whether O-GlcNAc-mediated activation of STING promotes IFN-associated antiviral response, we knocked down STING in KYSE-30 cells, reconstituted the cells with WT and T229A STING, and probed the expression of antiviral cytokines under HSV-1 stimulation. Depletion of STING in KYSE-30 cells resulted in a significant reduction in the transcript of genes encoding antiviral effectors, such as *Ifnb1*, *Il6*, *Tnfa*, *Isg15*, *Cxcl10*, and *Mx1* (Fig. 6A). Consistently, STING-depleted KYSE-30 cells produced a lower level of IFN-β and IL-6 as compared with those in control KYSE-30 cells (Fig. 6B, C). Under the challenge of HSV-1 or poly(dA:dT) for 16 h, shSTING KYSE-30 cells showed a markedly decreased expression of cytokines, such as *Ifnb1*, *Il6*, *Tnfa*, *Isg15*, *Cxcl10*, and *Mx1* (Fig. 6A), as well as IFN-β and IL-6 as compared with ctrl-shRNA-transduced cells stimulated with HSV-1 or poly(dA:dT) (Fig. 6B, C). Through a lentiviral vector-based transduction strategy, we reconstituted STING-knocked down cells with WT STING and found that the recombination fully restored cytokine production in the transcript (Fig. 6A) and protein (Fig. 6B, C) levels. However, STING-knocked down cells reconstituted with STING T229A mutant failed to restored cytokine production either at the mRNA or at the protein level regardless of the existence of HSV-1 or poly(dA:dT) (Fig. 6A-C). To further prove that mutation of STING T229 to alanine abrogated STING activation and reduced HSV-1 stimulated production of interferon (IFN). We constructed a STING mutant T229E, which depletes glycosylation and mimics phosphorylation at this site. As compared with WT STING-transduced cells, STING T229E mutant-transduced KYSE-30 cells bore a low level of *Ifnb1*, *Il6*, *Tnfa*, *Isg15*, *Cxcl10*, and *Mx1*, demonstrating an essential role of STING T229 O-GlcNAcylation in the activation of IFN antiviral immune signaling and production of inflammatory cytokines (Additional file 3: Fig. S4). In sum, these findings demonstrate an essential role of STING O-GlcNAcylation in the activation of IFN antiviral immune signaling and production of inflammatory cytokines.

### O-GlcNAcylation promotes antiviral immune response in mice

**Fig. 7:**
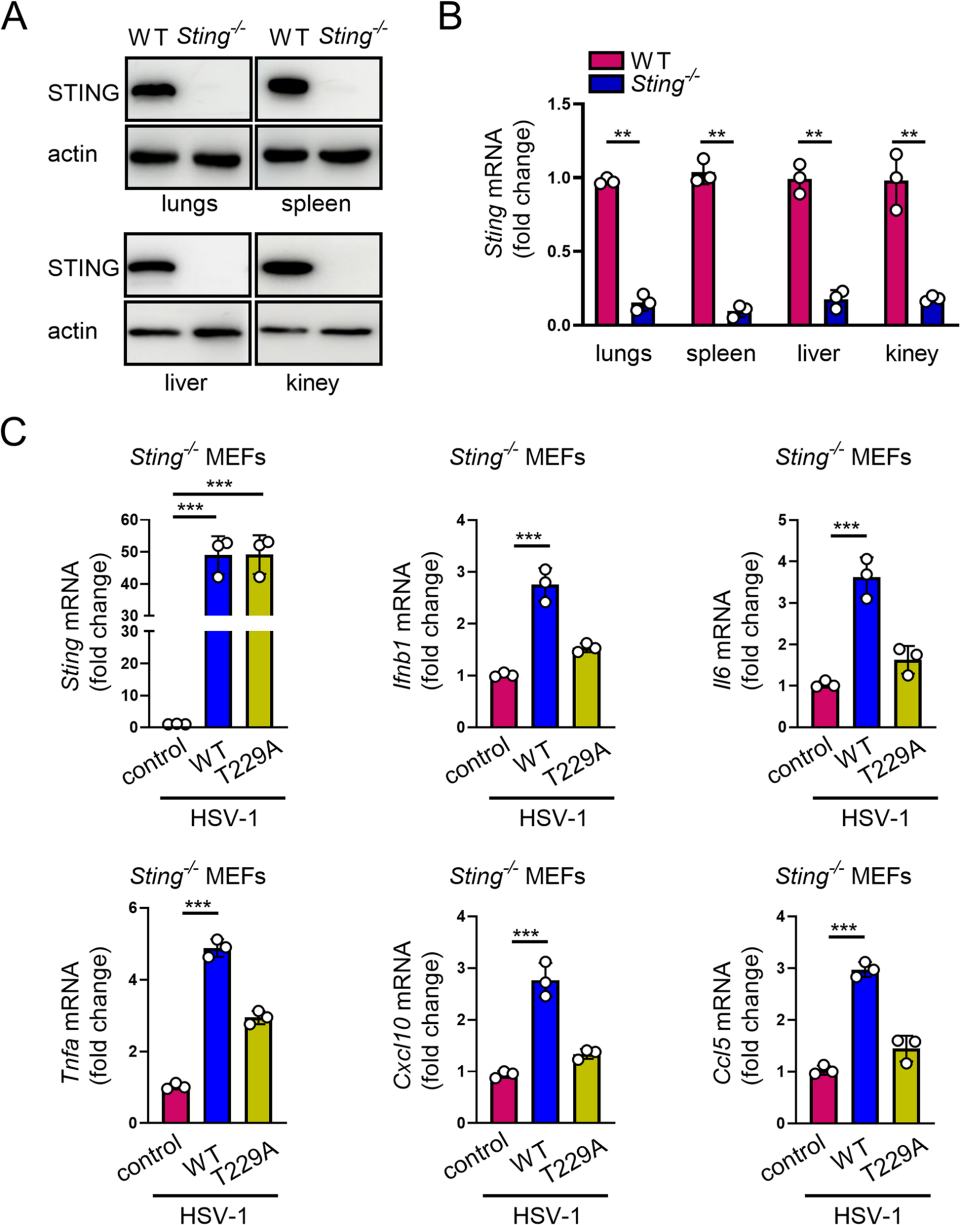
O-GlcNAcylation of STING activates antiviral immune response in MEFs. **A** and **B** Sting1^−/−^ mice were characterized by WB and qPCR. **C** Primary MEFs from *Sting1*.^−/−^ mice were reconstituted with WT or T229A STING. MEFs were then infected with HSV-1 (MOI = 1) for 16 h and used for detecting the transcript of *Ifnb1*, *Il6*, *Tnfa, Cxcl10,* and *Ccl5*. Data are representative of 3 independent biological replicates. Data are means ± SEM. ** *p* < 0.001, *** *p* < 0.0001

To explore whether O-GlcNAcylation of STING contributes to its known physiological functions in mice, we constructed *Sting1* knockout mice with CRISPR/Cas9 system. The establishment of *Sting* gene knockout mice was confirmed by qRT-PCR and immunoblot (Fig. 7A, B). Prior to validate the role of STING O-GlcNAc in antiviral response in mice, we isolated MEFs from *Sting1*^*−/−*^ mice and reconstituted these MEFs with either STING WT or T229A constructs through a lenti-vector-based transduction strategy. When the HSV-1 was introduced, *Sting1*^−/−^ MEFs reconstituted with WT STING restored the ability to activate IFN signaling, whereas those reconstituted with STING T229A mutation failed to do so (Fig. 7C), suggesting that STING O-GlcNAc contributes to HSV-1-stimulated generation of T1IFN in MEFs.

**Fig. 8:**
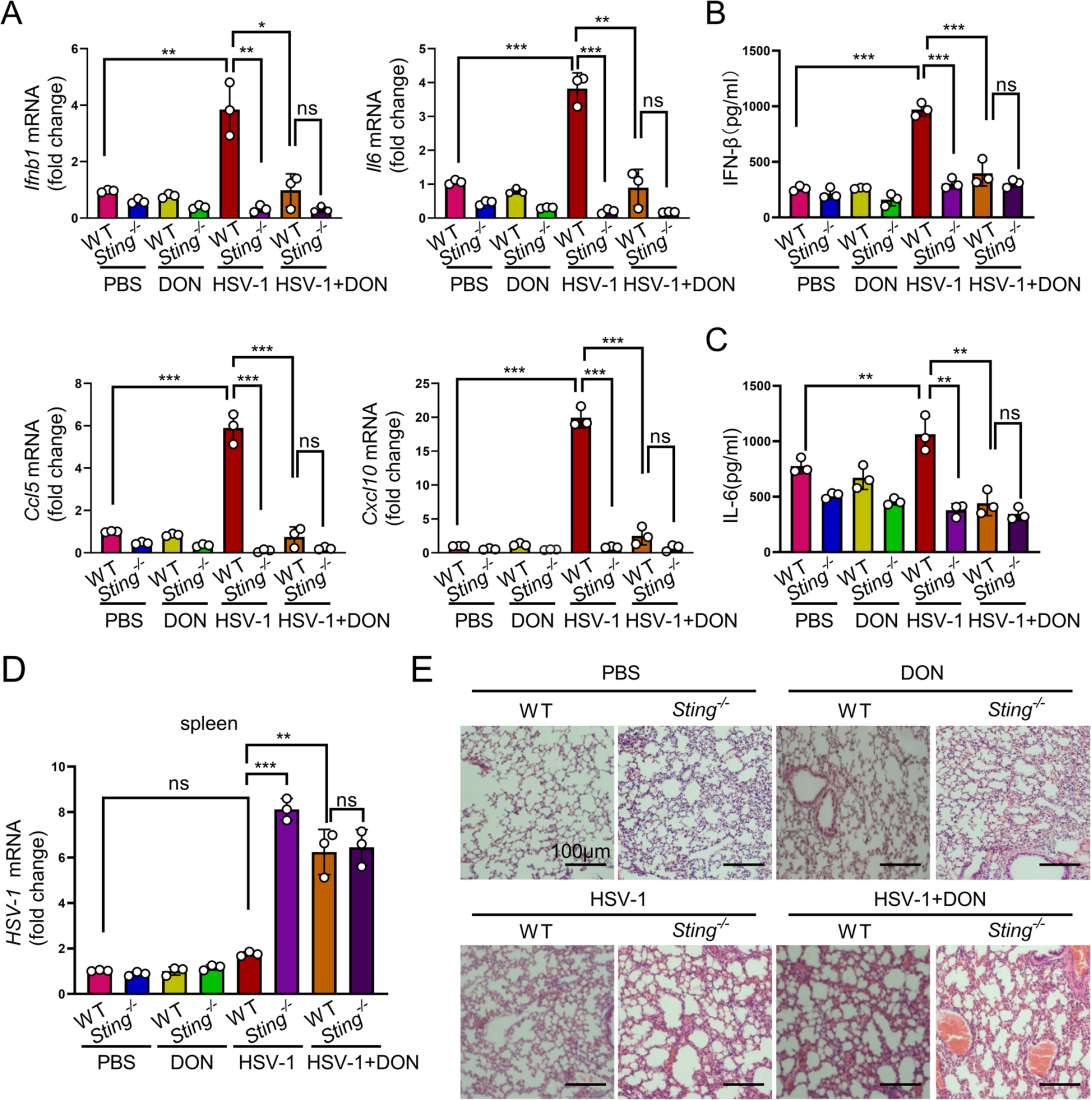
O-GlcNAcylation promotes antiviral immune response in vivo. **A** mRNAs of *Ifnb1*, *Il6*, *Ccl5*, and *Cxcl10* in the spleen of WT and *Sting1*^−/−^ mice challenged with HSV-1 (5 × 10^6^ PFU / mouse) for 6 days in the presence or absence of DON (1 mg/kg) (*n* = 6). **B** and **C** Levels of IFN-β and IL6 in serum from WT and *Sting1*^−/−^ mice challenged with HSV-1 (5 × 10^6^ PFU/mouse) for 6 days in the presence or absence of DON (1 mg/kg) (*n* = 6). **D** and **E** HSV-1 RNA in the spleen (**D**) and histological analysis of the lung tissue in WT and Sting1^−/−^ mice challenged with HSV-1 (5 × 10.^6^ PFU/mouse) for 6 days (*n* = 6), scale bar = 100 µm (**E**). Data are representatives of 3 independent biological replicates. Data are means ± SEM. NS, *p* > 0.05, * *p* < 0.05, ** *p* < 0.001. *** *p* < 0.0001

Next, we tested HSV-1 infection in *Sting1*^*−/−*^ and WT mice with or without O-GlcNAcylation manipulations. HSV-1 is a neurotropic DNA virus that is transmitted via mucosal tissues (typically oral, ocular, or genital) and infects epithelial cells before reaching the central nervous system where it establishes latency in neurons [36, 37]. To determine the role of STING O-GlcNAc in controlling HSV-1 infection, we intravenously injected HSV-1 into WT and *Sting1*^−/−^ mice and treated the mice with O-GlcNAc inhibitor DON or PBS. We detected the IFN antiviral immune signaling and the production of inflammatory cytokines in HSV-1-treated mice. RT-PCR analysis revealed a robust induction of *Ifnb1*, *Il6*, *Ccl5*, and *Cxcl10* gene transcription in the spleen from HSV-1-infected WT mice (Fig. 8A), indicating an effective induction of antiviral immunity. Cytokine expression as well as serum IFN-β and IL-6 were also significantly increased in HSV-1-infected WT mice (Fig. 8B, C). In contrast, HSV-1-infected WT mice with DON treatment bore a reduced cytokine expression and serum IFN-β and IL-6 levels (Fig. 8A-C). In addition, *Sting1*^−/−^ mice contained significantly blunted levels of cytokine expression and serum IFN-β and IL-6 regardless of the presence or absence of HSV-1 and/or DON treatment (Fig. 8A-C). Together, our data suggest that STING and its O-Glycosylation are critical for the antiviral response of the mice.

We further found that *Sting1*^−/−^ mice contained significantly higher levels of HSV-1 transcript in the spleen, liver, lung, and kidney, indicating an ineffective control of viral replication in Sting depleted mice (Fig. 8D and Additional file 3: Fig. S5A—C). Histological analysis of the lung, liver, and kidney tissues showed a greater infiltration of inflammatory cells and damages in HSV-1-infected *Sting1*^−/−^ mice compared with those in HSV-1-infected WT mice. Treatment with DON exacerbated the phenotypes of HSV-1-infected WT and eliminated the differences between WT and *Sting1*^−/−^ in response to HSV-1 challenge (Fig. 8E and Additional file 3: Fig. S5D, E). In addition, we also observed that HSV-1 infection led to an increase in the level of STING protein and O-Glycosylation in the lung (Additional file 3: Fig. S6) or liver (Additional file 3: Fig. S7), which was suppressed in mice exposed to DON, further demonstrating the importance of O-Glycosylation in the mediation of antiviral infection. Taken together, our data suggest that STING is crucial for the induction of antiviral innate immune response. Inhibition of O-GlcNAc attenuates STING-mediated removal of HSV-1 and increases infiltration of inflammatory cells and tissue damages in mice.

## Discussion

Metabolic reprogramming toward increased glucose uptake, glycolysis, and PPP has been well defined in innate immune cells that are classically activated by viral or bacterial infection [27]. For example, studies showed that elevated aerobic glycolysis upon viral infection is important for antiviral immune responses in mouse and human cells [18, 57]. HBP is a branch from the glycolysis pathway and its endpoint product, UDP-GlcNAc, provides donor glucosamine for glycosylation. O-GlcNAcylation has been reported to control a wide range of cellular functions [58]. A previous report showed that O-GlcNAcylation regulates the function of MAVS and highlights the importance of the PTM in antiviral innate immunity [18]. Hu et al. found that HBV infection upregulates GLUT1 expression and increases UDP-GlcNAc biosynthesis and O-GlcNAcylation in mice [59]. These findings demonstrate an essential function of O-GlcNAcylation in eliciting a robust host innate immune responses.

In the current study, we found that levels of metabolites in HBP, in particular UDP-GlcNAc, were elevated in HSV-1-infected cells (Fig. 1I). STING, a key signal transduction molecule of innate immune response triggered by DNA virus, was O-GlcNAcylated. More importantly, we demonstrated that O-GlcNacylation of STING was critical in eliciting the antiviral activity (Figs. 5 and 6). Although we could not completely rule out the existence of additional O-GlcNAcylation site(s) on STING, T229A mutation led to a striking suppression of O-GlcNAcylation in STING, indicating the importance of T229 in STING O-GlcNAcylation. O-GlcNAcylation of STING at T229 was required for HSV-1-induced IRF3 phosphorylation and IFN-β production (Figs. 5, 6, and 7), supporting the notion that regulation of STING O-GlcNAc could be a potential therapeutic strategy for DNA-virus infections. Wu et al. generated the *Sting1*^S365A/S365A^ mutant mouse that precisely ablates IFN-dependent activities whereas IFN-independent activities of STING are maintained. *Sting1*^S365A/S365A^ mice retain the activity against HSV-1 infection, despite lacking the STING-mediated IFN response [60]. Recent studies have shown that O-GlcNAcylation and phosphorylation exert an extensive crosstalk and mutually regulate cellular pathways and functions [57]. It remains unknown whether O-GlcNAcylation of STING at T229 affects the phosphorylation of T229 or neighboring residues or whether it retains its role in antiviral immune response in the background of *Sting1*^S365A/S365A^ mutation. We constructed a STING mutant T229E, which depletes glycosylation and mimics phosphorylation at this site. As compared with WT STING-transduced cells, STING T229E mutant-transduced KYSE-30 cells bore a low level of *Ifnb1*, *Il6*, *Tnfa*, *Isg15*, *Cxcl10*, and *Mx1*, demonstrating an essential role of STING T229 O-GlcNAcylation in the activation of IFN antiviral immune signaling and production of inflammatory cytokines (Additional file 3: Fig. S4).

It was reported that ubiquitination regulates the activities of STING in innate immune signaling. At least three E3 ubiquitin ligases have been confirmed to play a positively regulatory role in the activation of STING. E3 ubiquitin ligases TRIM32 and TRIM56 promote K63 polyubiquitination of STING, enhancing the activation of its downstream signaling [9, 10]. In the ER, the E3 ubiquitin ligase complex composed of AMFR-GP78 and INSIG1 promotes K27 polyubiquitination of STING, leading to the recruitment of TBK1 and production of interferon [11]. In contrast, the E3 ubiquitin ligases RNF5 and TRIM30a promote the K48 polyubiquitination of STING, resulting in its degradation by the proteasome and hence the inhibition of antiviral response [12]. In conclusion, ubiquitination modification plays multiple roles in regulating STING in a contextual dependent manner. In the current study, we found that induction of O-GlcNAcylation increased K63 ubiquitination of STING, whereas suppression of O-GlcNAcylation bore an opposite effect (Fig. 4D). O-GlcNAc of STING enhanced TRIM56 recruitment, increased K63-mediated ubiquitination of STING, and hence promoted the activation of STING (Additional file 3: Fig. S2D). In contrast, K63-linked ubiquitination of STING was evidently reduced in STING T229A mutant (Fig. 4E), further supporting the importance of STING T229 O-GlcNAcylation in the ubiquitination of STING and subsequent activation of antiviral innate immune. In terms of additional ubiquitination modifications of STING, we found that there was no difference in K27-linked ubiquitination in STING T229A as compared with that in WT STING (Additional file 3: Fig. S2D). It remains unknown whether O-GlcNAcylation of STING on T229 weakens the K48 polyubiquitination of STING, prevents it from proteasome-mediated degradation, and hence promotes the activation of antiviral signaling cascade. In the future, it is interesting to determine the impact of O-GlcNAcylation on K48 ubiquitination of STING and elucidate the functional connection of their possible crosstalk in STING-mediated antiviral immune response.

## Conclusion

**Fig. 9:**
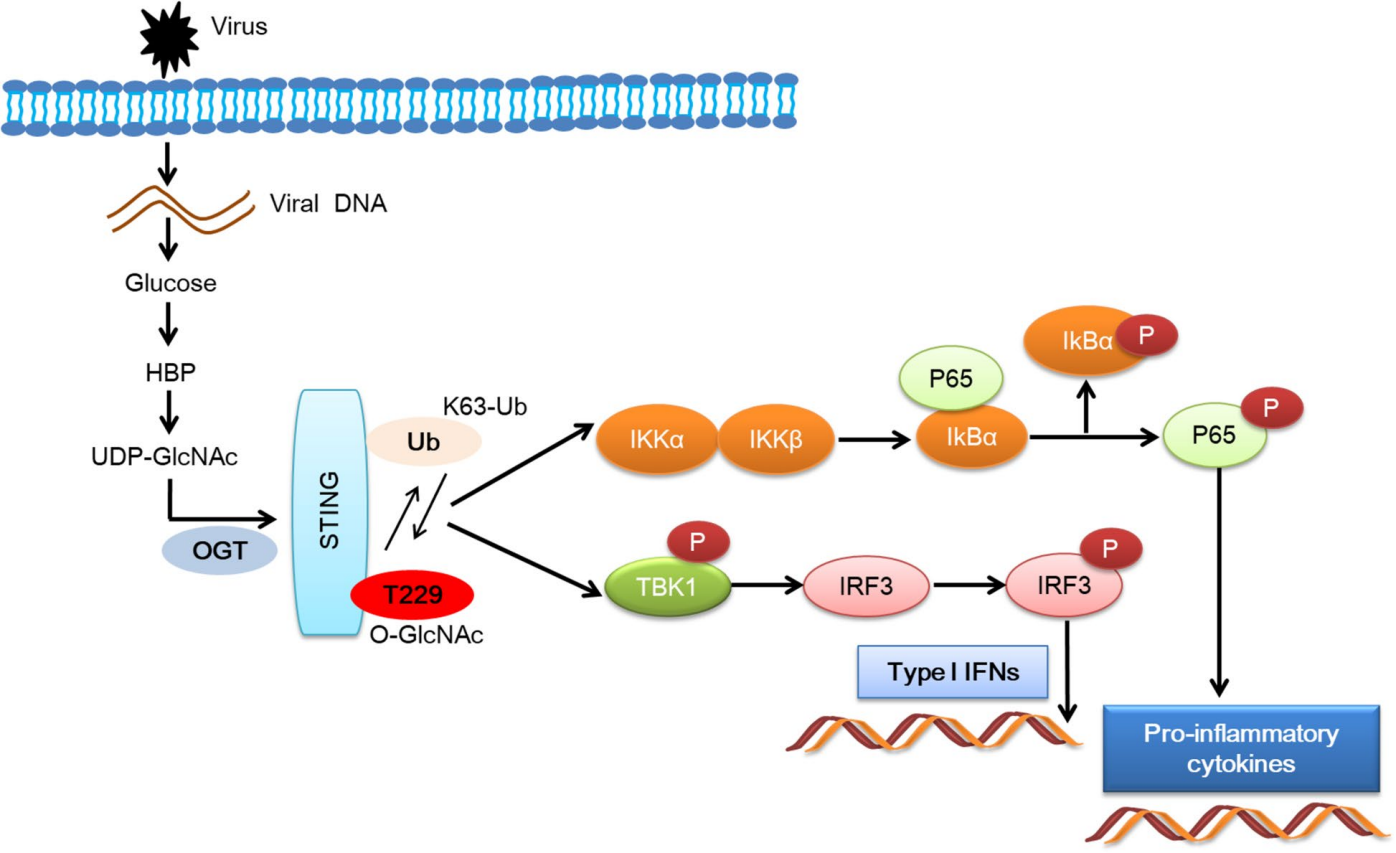
O-GlcNAcylation of STING mediates antiviral innate immunity

Elevated influx of glucose into the HBP promoted antiviral innate immune by enhancing STING O-GlcNAcylation. Current findings expand our understanding in the importance of glucose metabolism in viral infection-associated diseases (Fig. 9).

### Supplementary Information

**Additional file 1.** Supplementary sequences of primers and shRNAs.

**Additional file 2.** Supplementary reagents and antibodies used in this study.

**Additional file 3.** **Figure S1.** (A). Immunoblotting of phosphorylated IRF3 (p-IRF3), IRF3, OGT, and actin in shCtrl and shOGT cells infected with ISD (2 μg/mL) for 16 hrs. (B). Immunoblotting of phosphorylated IRF3 (p-IRF3), IRF3, OGT, and actin in shCtrl and shOGT cells infected with poly(dA:dT) (2 μg/mL) for 16 hrs. Data are representatives from 3 independent experiments. **Figure S2.** (A). O-GlcNAcylated proteins in KYSE-30 cells were pulled down with sWGA beads. cGAS was detected with an anti-cGAS antibody from abcam. (B). Immunoblotting of K63 ubiquitination in immunoprecipitated complex pulled down with STING antibody from lysates of cells treated with or without DON (10 μM) for 12 hrs. STING was immunoprecipitated with anti-STING antibody from abcam. OGT, and STING in the pulldown complex and in the input were detected with immunoblotting. (C). STING was immunoprecipitated with anti-STING antibody from abcam. K63-Ub, OGT, and STING in the pulldown complex and in the input were detected with immunoblotting. (D). HEK-293T cells were transfected with FLAG-tagged STING-WT or -T229A. Co-IP was performed with an anti-STING antibody from abcam. Immunoblotting was performed with antibodies against K27-Ub, K63-Ub, RL2, TRIM56 and STING. Data are representatives from 3 independent experiments. **Figure S3.** (A). Analyses of STING oligomerization by native gel electrophoresis. cGAMP (9 μg/mL, 16 h) was used to induce high-order oligomerization of STING. The results shown are representatives of three biological repeats. (B). KYSE-30 cells were transfected with FLAG-tagged STING-WT or -T229A and treated with 2 μg/mL ISD for 16 hrs. Cells were then fixed for immunofluorescence detection of STING and calnexin (ER marker) or GM130 (Golgi marker). Data are representatives from 3 independent experiments. **Figure S4.** KYSE-30 cells reconstituted with either STING-WT or STING-T229E were transfected with 2 μg/mL poly(dA:dT) by Lipofectamine 2000 for 16 hrs. Levels of Ifnb1, Il6, Tnfa, Isg15, Cxcl10, and Mx1 mRNA in cells were measured with RT-PCR. Data from representatives of 3 independent biological replicates are presented. Data are means ± SEM. ** *p* < 0.001, *** *p* < 0.0001. **Figure S5.** (A-C). Detection of HSV-1 in the liver, lung, and kidney from WT and Sting1-/- mice challenged with HSV-1 (5 x 106 PFU/mouse) for 6 days (*n* = 6). (D). Detection of liver tissue injury in WT and Sting1-/- mice pretreated with or without DON and challenged with either PBS or HSV-1 (5 x 106 PFU/mouse) for 6 days (*n* = 6 per group), scale bar = 100 µm. (E). Detection of kidney tissue injury in WT and Sting1-/- mice pretreated with or without DON and challenged with either PBS or HSV-1 (5 x 106 PFU/mouse) for 6 days (*n* = 6 per group), scale bar = 100 µm. Data from representatives of 3 independent biological replicates are presented. Data are means ± SEM. NS, *p* > 0.05, *** *p* < 0.0001. **Figure S6.** Immunohistochemical staining was applied for the detection of STING and total O-GlcNAc modification in lungs from WT and Sting1-/- mice pretreated with or without DON and challenged with either PBS or HSV-1 (5 x 106 PFU/mouse) for 6 days (*n* = 6 per group), scale bar = 100 µm. Data are representatives of 3 independent biological replicates. Data are means ± SEM. NS, *p* > 0.05, * *p* < 0.05, **p < 0.001, *** *p* < 0.0001. **Figure S7.** Immunohistochemical staining was applied for the detection of STING and total O-GlcNAc modification in livers from WT and Sting1-/- mice pretreated with or without DON and challenged with either PBS or HSV-1 (5 x 106 PFU/mouse) for 6 days (*n* = 6 per group). Data are representatives of 3 independent biological replicates. Data are means ± SEM. NS, *p* > 0.05, * *p* < 0.05, ** *p* < 0.001, *** *p* < 0.0001.

## Data Availability

The datasets generated during this study are available in the Dryad repository:

## Abbreviations

STING: Stimulator of interferon genes
HBP: Hexosamine biosynthesis pathway
DON: 6-Diazo-5-oxonorleucine
TMG: Thiamet-G
T1IFNs: Type I interferons
ISGs: IFN-stimulated genes
HSV-1: Herpes simplex virus-1
Poly(dA:dT): Poly (deoxyadenylic-deoxythymidylic) acid sodium salt
Poly(I:C): Polyinosinic-polycytidylic acid
MEFs: Mouse embryonic fibroblasts
ISD: Interferon stimulatory DNA
cGAMP: Cyclic GMP-AMP; 3',3'-Cgamp

## References

[1] Sato A, Linehan MM, Iwasaki A (2006). Dual recognition of herpes simplex viruses by TLR2 and TLR9 in dendritic cells. Proc Natl Acad Sci USA.

[2] Watson RO, Bell SL, MacDuff DA, Kimmey JM, Diner EJ, Olivas J, Vance RE, Stallings CL, Virgin HW, Cox JS (2015). The cytosolic sensor cGAS detects mycobacterium tuberculosis DNA to induce Type I interferons and activate autophagy. Cell Host Microbe.

[3] Watson RO, Manzanillo PS, Cox JS (2012). Extracellular M. tuberculosis DNA Targets Bacteria for Autophagy by Activating the Host DNA-Sensing Pathway. Cell.

[4] Yamashiro LH, Wilson SC, Morrison HM, Karalis V, Chung JYJ, Chen KJ, Bateup HS, Szpara ML, Lee AY, Cox JS, Vance RE (2020). Interferon-independent STING signaling promotes resistance to HSV-1 in vivo. Nat Commun.

[5] Motwani M, Pesiridis S, Fitzgerald KA (2019). DNA sensing by the cGAS-STING pathway in health and disease. Nat Rev Genet.

[6] Tan X, Sun L, Chen J, Chen ZJ (2018). Detection of microbial infections through innate immune sensing of nucleic acids. Annu Rev Microbiol.

[7] Shang G, Zhang C, Chen ZJ, Bai XC, Zhang X (2019). Cryo-EM structures of STING reveal its mechanism of activation by cyclic GMP-AMP. Nature.

[8] Zhang C, Shang G, Gui X, Zhang X, Bai XC, Chen ZJ (2019). Structural basis of STING binding with and phosphorylation by TBK1. Nature.

[9] Seo GJ, Kim C, Shin WJ, Sklan EH, Eoh H, Jung JU (2018). TRIM56-mediated monoubiquitination of cGAS for cytosolic DNA sensing. Nat Commun.

[10] Zhang J, Hu MM, Wang YY, Shu HB (2012). TRIM32 Protein modulates Type I interferon induction and cellular antiviral response by targeting MITA/STING protein for K63-linked ubiquitination. J Biol Chem.

[11] Wang Q, Liu X, Cui Y, Tang YJ, Chen W, Li SL, Yu HS, Pan YD, Wang C (2014). The E3 ubiquitin ligase AMFR and INSIG1 bridge the activation of TBK1 Kinase by modifying the adaptor STING. Immunity.

[12] Wang YM, Lian QS, Yang B, Yan SS, Zhou HY, He L, Lin GM, Lian ZX, Jiang ZF, Sun B (2015). TRIM30 alpha Is a Negative-feedback regulator of the intracellular DNA and DNA virus-triggered response by targeting STING. PLoS Pathog.

[13] Yang LL, Xiao WC, Li H, Hao ZY, Liu GZ, Zhang DH, Wu LM, Wang Z, Zhang YQ, Huang Z, Zhang YZ (2022). E3 ubiquitin ligase RNF5 attenuates pathological cardiac hypertrophy through STING. Cell Death Dis.

[14] Yum S, Li M, Fang Y, Chen ZJ (2021). TBK1 recruitment to STING activates both IRF3 and NF-κB that mediate immune defense against tumors and viral infections. Proc Natl Acad Sci U S A.

[15] Zhang WN, Wang GH, Xu ZG, Tu HQ, Hu FQ, Dai J, Chang Y, Chen YQ, Lu YJ, Zeng HL (2019). Lactate Is a Natural Suppressor of RLR Signaling by Targeting MAVS. Cell.

[16] Wenes M, Shang M, Di Matteo M, Goveia J, Martin-Perez R, Serneels J, Prenen H, Ghesquiere B, Carmeliet P, Mazzone M (2016). Macrophage metabolism controls tumor blood vessel morphogenesis and metastasis. Cell Metab.

[17] Lu Y, Li Y, Liu Q, Tian N, Du P, Zhu F, Han Y, Liu X, Liu X, Peng X (2021). MondoA-thioredoxin-interacting protein axis maintains regulatory T-Cell identity and function in colorectal cancer microenvironment. Gastroenterology.

[18] Li T, Li X, Attri KS, Liu C, Li L, Herring LE, Asara JM, Lei YL, Singh PK, Gao C, Wen H (2018). O-GlcNAc Transferase links glucose metabolism to MAVS-mediated antiviral innate immunity. Cell Host Microbe.

[19] Yang XY, Qian KV (2017). Protein O-GlcNAcylation: emerging mechanisms and functions. Nat Rev Mol Cell Biol.

[20] Hardiville S, Hart GW (2014). Nutrient regulation of signaling, transcription, and cell physiology by O-GlcNAcylation. Cell Metab.

[21] Levine ZG, Walker S (2016). The biochemistry of O-GlcNAc Transferase: which functions make it essential in mammalian cells?. Annu Rev Biochem.

[22] Lu S, Liao ZD, Lu XY, Katschinski DM, Mercola M, Chen J, Brown JH, Molkentin JD, Bossuyt J, Bers DM (2020). Hyperglycemia acutely increases cytosolic reactive oxygen Species via O-linked GlcNAcylation and CaMKII activation in mouse ventricular myocytes. Circ Res.

[23] Covert JD, Grice BA, Thornburg MG, Kaur M, Ryan AP, Tackett L, Bhamidipati T, Stull ND, Kim T, Habegger KM (2023). An early, reversible cholesterolgenic etiology of diet-induced insulin resistance. Molecular Metabolism.

[24] Zuliani I, Lanzillotta C, Tramutola A, Barone E, Perluigi M, Rinaldo S, Paone A, Cutruzzola F, Bellanti F, Spinelli M (2021). High-fat diet leads to reduced protein O-GlcNAcylation and mitochondrial defects promoting the development of alzheimer's disease signatures. Int J Mol Sci.

[25] Swamy M, Pathak S, Grzes KM, Damerow S, Sinclair LV, Van Aalten DM, Cantrell DA (2016). Glucose and glutamine fuel protein O-GlcNAcylation to control T cell self-renewal and malignancy. Nat Immun.

[26] Liu B, Salgado OC, Singh S, Hippen KL, Maynard JC, Burlingame AL, Ball LE, Blazar BR, Farrar MA, Hogquist KA, Ruan HB (2019). The lineage stability and suppressive program of regulatory T cells require protein O-GlcNAcylation. Nat Commun.

[27] Shi Q, Shen Q, Liu Y, Shi Y, Huang W, Wang X, Li Z, Chai Y, Wang H, Hu X (2022). Increased glucose metabolism in TAMs fuels O-GlcNAcylation of lysosomal Cathepsin B to promote cancer metastasis and chemoresistance. Cancer Cell.

[28] Wu JL, Chiang MF, Hsu PH, Tsai DY, Hung KH, Wang YH, Angata T, Lin KI (1854). O-GlcNAcylation is required for B cell homeostasis and antibody responses. Nat Commun.

[29] Ramakrishnan P, Clark PM, Mason DE, Peters EC, Hsieh-Wilson LC, Baltimore D (2013). Activation of the transcriptional function of the NF-kappaB protein c-Rel by O-GlcNAc glycosylation. Sci Signal.

[30] Zhao M, Ren K, Xiong X, Cheng M, Zhang Z, Huang Z, Han X, Yang X, Alejandro EU, Ruan HB (2020). Protein O-GlcNAc modification links dietary and gut microbial cues to the differentiation of enteroendocrine L cells. Cell Rep.

[31] Liu YY, Liu HY, Yu TJ, Lu Q, Zhang FL, Liu GY, Shao ZM, Li DQ (2022). O-GlcNAcylation of MORC2 at threonine 556 by OGT couples TGF-beta signaling to breast cancer progression. Cell Death Differ.

[32] Park SJ, Bae JE, Jo DS, Kim JB, Park NY, Fang J, Jung YK, Jo DG, Cho DH (2021). Increased O-GlcNAcylation of Drp1 by amyloid-beta promotes mitochondrial fission and dysfunction in neuronal cells. Mol Brain.

[33] Johnsen VLBD, Hughey CC, Hittel DS, Hepple RT, Koch LG, Britton SL, Shearer J (2013). Enhanced cardiac protein glycosylation (O-GlcNAc) of selected mitochondrial proteins in rats artificially selected for low running capacity. Physiol Genomics.

[34] Bao DK, Zhao J, Zhou XC, Yang Q, Chen YB, Zhu JJ, Yuan P, Yang J, Qin T, Wan SG, Xing JL (2019). Mitochondrial fission-induced mtDNA stress promotes tumor-associated macrophage infiltration and HCC progression. Oncogene.

[35] Liu BY, Zhang M, Chu HL, Zhang HH, Wu HF, Song GH, Wang P, Zhao K, Hou JX, Wang X (2017). The ubiquitin E3 ligase TRIM31 promotes aggregation and activation of the signaling adaptor MAVS through Lys63-linked polyubiquitination. Nat Immunol.

[36] Nicoll MP, Proenc JT, Efstathiou S (2012). The molecular basis of herpes simplex virus latency. FEMS Microbiol Rev.

[37] Steiner I, Benninger F (2013). Update on herpes virus infections of the nervous system. Curr Neurol Neurosci Rep.

[38] Zhang L, Wei X, Wang Z, Liu P, Hou Y, Xu Y, Su H, Koci MD, Yin H, Zhang C (2023). NF-kappaB activation enhances STING signaling by altering microtubule-mediated STING trafficking. Cell Rep.

[39] Jimenez-Gonzalez M, Li R, Pomeranz LE, Alvarsson A, Marongiu R, Hampton RF, Kaplitt MG, Vasavada RC, Schwartz GJ, Stanley SA (2022). Mapping and targeted viral activation of pancreatic nerves in mice reveal their roles in the regulation of glucose metabolism. Nat Biomed Eng.

[40] Codo AC, Davanzo GG, Monteiro LB, de Souza GF, Muraro SP, Virgilio-da-Silva JV, Prodonoff JS, Carregari VC, de Biagi Junior CAO, Crunfli F (2020). Elevated glucose levels favor SARS-CoV-2 infection and monocyte response through a HIF-1alpha/Glycolysis-Dependent Axis. Cell Metab.

[41] Wang A, Huen SC, Luan HH, Yu S, Zhang C, Gallezot JD, Booth CJ, Medzhitov R (2016). Opposing effects of fasting metabolism on tissue tolerance in bacterial and viral inflammation. Cell.

[42] Angelova M, Ortiz-Meoz RF, Walker S, Knipe DM (2015). Inhibition of O-Linked N-Acetylglucosamine transferase reduces replication of herpes simplex virus and human cytomegalovirus. J Virol.

[43] Ortiz-Meoz RF, Jiang JY, Lazarus MB, Orman M, Janetzko J, Fan CG, Duveau DY, Tan ZW, Thomas CJ, Walker S (2015). A small molecule that inhibits OGT activity in cells. ACS Chem Biol.

[44] Li X, Zhang Z, Li L, Gong W, Lazenby AJ, Swanson BJ, Herring LE, Asara JM, Singer JD, Wen H (2017). Myeloid-derived cullin 3 promotes STAT3 phosphorylation by inhibiting OGT expression and protects against intestinal inflammation. J Exp Med.

[45] Hart GW, Slawson C, Ramirez-Correa G, Lagerlof O (2011). Cross talk between O-GlcNAcylation and phosphorylation: roles in signaling, transcription, and chronic disease. Annu Rev Biochem.

[46] Takahashi M, Lio CJ, Campeau A, Steger M, Ay F, Mann M, Gonzalez DJ, Jain M, Sharma S (2021). The tumor suppressor kinase DAPK3 drives tumor-intrinsic immunity through the STING-IFN-beta pathway. Nat Immunol.

[47] Yang B, Pei J, Lu C, Wang Y, Shen M, Qin X, Huang Y, Yang X, Zhao X, Ma S (2023). RNF144A promotes antiviral responses by modulating STING ubiquitination. EMBO Rep.

[48] Gack MU, Qin Y, Zhou M-T, Hu M-M, Hu Y-H, Zhang J, Guo L, Zhong B, Shu H-B (2014). RNF26 Temporally Regulates Virus-Triggered Type I Interferon Induction by Two Distinct Mechanisms. PLoS Pathog.

[49] Fenech EJ, Lari F, Charles PD, Fischer R, Laétitia-Thézénas M, Bagola K, Paton AW, Paton JC, Gyrd-Hansen M, Kessler BM, Christianson JC (2020). Interaction mapping of endoplasmic reticulum ubiquitin ligases identifies modulators of innate immune signalling. Life.

[50] Zhang X (2020). Bai X-c, Chen ZJ: structures and mechanisms in the cGAS-STING innate immunity pathway. Immunity.

[51] Wu J, Sun L, Chen X, Du F, Shi H, Chen C, Chen ZJ (2013). Cyclic GMP-AMP is an endogenous second messenger in innate immune signaling by cytosolic DNA. Science.

[52] Sun L, Wu J, Du F, Chen X, Chen ZJ (2013). Cyclic GMP-AMP synthase is a cytosolic DNA sensor that activates the Type I interferon pathway. Science.

[53] Dobbs NBN, Chen D, Gonugunta VK, Alto NM, Yan N (2015). STING activation by translocation from the ER is associated with infection and autoinflammatory disease. Cell Host Microbe.

[54] Ogawa E, Mukai K, Saito K, Arai H, Taguchi T (2018). The binding of TBK1 to STING requires exocytic membrane traffic from the ER. Biochem Biophys Res Commun.

[55] Baatarjav C, Komada T, Karasawa T, Yamada N, Sampilvanjil A, Matsumura T, Takahashi M (2022). dsDNA-induced AIM2 pyroptosis halts aberrant inflammation during rhabdomyolysis-induced acute kidney injury. Cell Death And Differentiation.

[56] Wang C, Guan Y, Lv M, Zhang R, Guo Z, Wei X, Du X, Yang J, Li T, Wan Y (2018). Manganese increases the sensitivity of the cGAS-STING pathway for double-stranded DNA and is required for the host defense against DNA viruses. Immunity.

[57] Song N, Qi Q, Cao RY, Qin BJ, Wang B, Wang YX, Zhao L, Li W, Du XL, Liu F (2019). MAVS O-GlcNAcylation Is Essential for Host Antiviral Immunity against Lethal RNA Viruses. Cell Rep.

[58] Wu LM, Cheng YX, Geng DD, Fan ZY, Lin BY, Zhu Q, Li JC, Qin WJ, Yi W (2022). O-GlcNAcylation regulates epidermal growth factor receptor intracellular trafficking and signaling. Proc Natl Acad Sci USA.

[59] Hu J, Gao Q, Yang Y, Xia J, Zhang W, Chen Y, Zhou Z, Chang L, Hu Y, Zhou H (2021). Hexosamine biosynthetic pathway promotes the antiviral activity of SAMHD1 by enhancing O-GlcNAc transferase-mediated protein O-GlcNAcylation. Theranostics.

[60] Wu JJ, Dobbs N, Yang K, Yan N (2020). Interferon-Independent Activities of Mammalian STING Mediate Antiviral Response and Tumor Immune Evasion. Immunity.

