# Supplementary material for: O-GlcNAc of STING mediates antiviral innate immunity
Source: Cell Commun Signal. 2024 Mar 1;22:157. doi: 10.1186/s12964-024-01543-8 (PMC10908090; doi:10.1186/s12964-024-01543-8)
Supplement: Supplementary file 1 — Additional file 1. Supplementary sequences of primers and shRNAs. [file 12964_2024_1543_MOESM1_ESM.docx]

**Table S1 Sequences of primers and shRNAs**

| 1. **Primers used in PCR analyses** | | |
| --- | --- | --- |
| *Sting* Human | Forward primer | AGCATTACAACAACCTGCTACG |
|  | Reverse primer | GTTGGGGTCAGCCATACTCAG |
| *Ifnb1* Human | Forward primer | CAACAAGTGTCTGCTCGAAAT |
|  | Reverse primer | TCTCCTCAGGGATGTCAAAG |
| *Il6* Human | Forward primer | TGGCTGCAGGACATGACAACT |
|  | Reverse primer | ATCTGAGGTGCCCATGCTACA |
| *Tnfa* Human | Forward primer | GCGGGAAATATGACAGCTAAGG |
|  | Reverse primer | TGCTTGTCTGGAACAACTGC |
| *Isg15* Human | Forward primer | TGACTGTGAGAGCAAGCAGC |
|  | Reverse primer | CCCCAGCATCTTCACCTTTA |
| *Cxcl10* Human | Forward primer | GTGGCATTCAAGGAGTACCTC |
|  | Reverse primer | TGATGGCCTTCGATTCTGGATT |
| *Mx1* Human | Forward primer | CAATCAGCCTGCTGACATTG |
|  | Reverse primer | TGTCTCCTGCCTCTGGATG |
| *GAPDH* Human | Forward primer | AACGGATTTGGTCGTATTGG |
|  | Reverse primer | TTGATTTTGGAGGGATCTCG |
| *Sting* Mouse | Forward primer | CTCATTGTCTACCAAGAACC |
|  | Reverse primer | TAACCTCCTCCTTTTCTTCC |
| *HSV-1* | Forward primer | CATCACCGACCCGGAGAGGGAC |
|  | Reverse primer | GGGCCAGGCGCTTGTTGGTGTA |
| *Ifnb1* Mouse | Forward primer | CTGGCTTCCATCATGAACAA |
|  | Reverse primer | AGAGGGCTGTGGTGGAGAA |
| *Il6* Mouse | Forward primer | TAGTCCTTCCTACCCCAATTTCC |
|  | Reverse primer | TTGGTCCTTAGCCACTCCTTC |
| *Ccl5* Mouse | Forward primer | CAAGTGCTCCAATCTTGCAGTC |
|  | Reverse primer | TTCTCTGGGTTGGCACACAC |
| *Cxcl10* Mouse | Forward primer | ATCATCCCTGCGAGCCTATCCT |
|  | Reverse primer | GACCTTTTTTGGCTAAACGCTTTC |
| *GAPDH* Mouse | Forward primer | TCACCATCTTCCAGGAGCGAGAC |
|  | Reverse primer | AGACACCAGTAGACTCCACGACATAC |
| **2. Primer sequences for molecular cloning in recombinant DNAs.**   \| pcDNA3.1(+)-his Ub \| Forward primer \| ATGCACCACCATCACCATCAT \| \| --- \| --- \| --- \| \| Reverse primer \| CTAACCACCTCTCAGACG \| \| Lenti CRISPR v3-STING(WT) \| Forward primer \| GGACCGGTTCTAGAGCCACCATGCCCCACTCCAGCCTGCA \| \| Reverse primer \| TCTGGAACATCGTATGGGTAAGAGAAATCCGTGCGGAGAG \| \| Lenti CRISPR v3-STING(S4A/S5A) \| Forward primer \| GGACCGGTTCTAGAGCCACCATGCCCCACGCCGCCCTGCA \| \| Reverse primer \| TCTGGAACATCGTATGGGTAAGAGAAATCCGTGCGGAGAG \| \| Lenti CRISPR v3-STING(S5A) \| Forward primer \| GGACCGGTTCTAGAGCCACCATGCCCCACTCCGCCCTGCA \| \| Reverse primer \| TCTGGAACATCGTATGGGTAAGAGAAATCCGTGCGGAGAG \| \| Lenti CRISPR v3-STING(T229A) \| Forward primer \| GGATAAACTGCCCCAGCAGGCCGGTGACCATGCTGGCATC \| \| Reverse primer \| GATGCCAGCATGGTCACCGGCCTGCTGGGGCAGTTTATCC \| \| Lenti CRISPR v3-STING(S322A) \| Forward primer \| AACCTGCAGATGACAGCGCCTTCTCGCTGTCCCAG \| \| Reverse primer \| CTGGGACAGCGAGAAGGCGCTGTCATCTGCAGGTT \| \| Lenti CRISPR v3-STING(S305A) \| Forward primer \| TGGCAGATGCCCCTGAGGCCCAGAACAACTGCCGC \| \| Reverse primer \| GCGGCAGTTGTTCTGGGCCTCAGGGGCATCTGCCA \| \| Lenti CRISPR v3- STING(S195A) \| Forward primer \| ATCAGCATTATAACAACCTGCTACGGGGTGCAGTGGCCCAGC \| \| Reverse primer \| GCTGGGCCACTGCACCCCGTAGCAGGTTGTTATAATGCTGAT \| \| Lenti CRISPR v3-STING(ST354A) \| Forward primer \| CTCAGCGGTGCCCAGTGCCTCCACGATGTCCCAAG \| \| Reverse primer \| CTTGGGACATCGTGGAGGCACTGGGCACCGCTGAG \| \| Lenti CRISPR v3-STING(T348A/S349A) \| Forward primer \| GTGGGCAGCTTGAAGGCCGCCGCGGTGCCCAGTAC \| \| Reverse primer \| TCTGGAACATCGTATGGGTAGGCGAAATCGGCGCGGAGAG \| \| Lenti CRISPR v3-STING(S376A/S379A) \| Forward primer \| GGACCGGTTCTAGAGCCACCATGCCCCACTCCAGCCTGCA \| \| Reverse primer \| TCTGGAACATCGTATGGGTAGGCGAAATCGGCGCGGAGAG \|   **3. shRNA** | | |
| pGPU6-STING shRNA | GCATTACAACAACCTGCTACG | |
