# Supplementary material for: O-GlcNAc of STING mediates antiviral innate immunity
Source: Cell Commun Signal. 2024 Mar 1;22:157. doi: 10.1186/s12964-024-01543-8 (PMC10908090; doi:10.1186/s12964-024-01543-8)
Supplement: Supplementary file 2 — Additional file 2. Supplementary reagents and antibodies used in this study. [file 12964_2024_1543_MOESM2_ESM.docx]

**Table S2 Reagents and antibodies used in this study**

| **Product name** | **Manufacturer** | **Catalog No.** |
| --- | --- | --- |
| **Antibodies for Western blotting** | | |
| Rabbit monoclonal anti-STING (IB assay) | Cell Signaling Technology | Cat# 13647 |
| Rabbit monoclonal anti-STING (IB assay) | Novus Biologicals | Cat# NBP2-24683 |
| Rabbit monoclonal anti-STING (IP assay) | Abcam | Cat# ab288157 |
| Rabbit monoclonal anti-phospho-STING (Ser366) | Cell Signaling Technology | Cat# 19781 |
| Rabbit monoclonal anti-NAK/TBK1 | Cell Signaling Technology | Cat#3504 |
| Rabbit monoclonal anti-phospho-NAK/TBK1 (Ser 172) | Cell Signaling Technology | Cat#5483 |
| Rabbit polyclonal anti-IRF3 | Cell Signaling Technology | Cat#4962 |
| Rabbit monoclonal anti-phospho-IRF3 (Ser396) | Cell Signaling Technology | Cat#4947 |
| Rabbit monoclonal anti-OGT | Cell Signaling Technology | Cat#5368 |
| Rabbit monoclonal anti-OGA | Novus Biologicals | Cat# NBP1-81244 |
| Rabbit monoclonal anti-NF-kB p65 | Cell Signaling Technology | Cat# 8242 |
| Rabbit monoclonal anti-phospho-NF-kB p65 (Ser536) | Cell Signaling Technology | Cat# 3033 |
| Rabbit polyclonal anti-IkBα | Cell Signaling Technology | Cat# 9242S |
| Rabbit monoclonal anti-phospho-IkBα(Ser32) | Cell Signaling Technology | Cat# 2859 |
| Rabbit monoclonal anti-phospho-IKKα/β (Ser176/180) | Cell Signaling Technology | Cat# 2697 |
| Rabbit monoclonal anti-FLAG | Novus Biologicals | Cat# NBP1-06712SS |
| Mouse monoclonal anti- GFPT1 | Abcam | Cat#ab125069 |
| Rabbit monoclonal anti-His-peroxidase | Proteintech | Cat# 10001-0-AP |
| Rabbit monoclonal anti-K63-linkage poly ubiquitin | Abcam | Cat# ab179434 |
| Rabbit monoclonal anti-K27-linkage polyubiquitin | Abcam | Cat# ab181537 |
| Mouse monoclonal anti-STING (IB assay) | Proteintech | Cat# 66680-1-1g |
| Rabbit polyclonal anti-GOLGA2/GM130 | Proteintech | Cat# 11308-1-AP |
| Rabbit monoclonal anti-TRIM56 | Zenbio | Cat# R25985 |
| Rabbit polyclonal anti-Calnexin | Proteintech | Cat# 10427-2-AP |
| Mouse monoclonal Anti-O-linked N-Acetylglucosamine [RL2] | Novus Biologicals | Cat# NBP2-59309 |
| Mouse monoclonal anti-actin | Proteintech | Cat# 60008-1-Ig |
| **Antibodies for Immunohistochemistry (IHC)** | | |
| Rabbit monoclonal anti-STING | Novus Biologicals | Cat# NBP2-24683 |
| Mouse monoclonal Anti-O-linked N-Acetylglucosamine [RL2] | Novus Biologicals | Cat# NBP2-59309 |
| **Secondary Antibodies** | | |
| Anti-mouse IgG, HRP-linked Antibody | Proteintech | Cat# SA00001-1 |
| Anti-rabbit IgG, HRP-linked Antibody | Proteintech | Cat# SA00001-2 |
| Biological Samples |  |  |
| *Sting1^-/-^* Mouse samples | This paper | N/A |
| **Chemicals, Peptides, and Recombinant Proteins** | | |
| Poly(I:C) | Invivogen | Cat# tlrl-pic-5 |
| Poly(dA:dT) | Invivogen | Cat# tlrl-patn-1 |
| ISD | Invivogen | Cat# tlrl-isdn |
| 3'3'-cGAMP | Invivogen | Cat# tlrl-nacga |
| Lipofectamine 2000 | Invitrogen | Cat# 11668019 |
| RIPA buffer | Beyotime, | Cat# P0013B |
| TRIzol LS Reagent | Invitrogen | Cat# 10296010 |
| Phosphatase Inhibitor Cocktail | Roche | Cat# 04906845001 |
| Protease Inhibitor Cocktail | Roche | Cat# 04693159001 |
| Geneticin (G-418 Sulfate) | Invitrogen | Cat# 10131027 |
| Thiamet G | MCE | Cat# HY-12588 |
| L-6-Diazo-5-oxonorleucine; DON | MCE | Cat# HY-108357 |
| succinylated Wheat Germ Agglutinin, sWGA | Vector group | Cat# AL-1023S-2 |
| Herpes Simplex Virus 1, HSV-1 | GENE | Cat# HSV-H129-G4 |
| Protein A/G | Cell Signaling Technology | Cat# 37478 |
| **Critical Commercial Assay Kits** | | |
| Instant Immunohistochemistry Kit | Sangon | Cat# C516337 |
| BCA Protein Assay Kit | Solarbio | Cat# PC0020 |
| CellAmp™ Direct SYBR®RT-qPCR Kit | TaKaRa | Cat# 3735A |
| PrimeScript™RT Master Mix (Perfect Real Time) | TaKaRa | Cat# RR036Q |
| Tissue DNA Kit | Solarbio | Cat# D1700 |
| ELISA IFN-β human | mlbio | Cat# YJ710284 |
| ELISA IL-6 human | mlbio | Cat# YJ028583 |
| ELISA IFN-β Mouse | mlbio | Cat# YJ720131 |
| ELISA IL-6 Mouse | mlbio | Cat# YJ063159 |
| UDP-GlcNAc-Mouse | mlbio | Cat# JL45954 |
| **Experimental Models: Cell Lines** | | |
| KYSE-30 | This paper | N/A |
| HEK-293T | This paper | N/A |
| *Sting1^-/-^* MEFs | This paper | N/A |
| **Oligonucleotides** | | |
| Primers for cloning, see Table S1 | This paper | N/A |
| Primers for qPCR, see Table S1 | This paper | N/A |
| shRNA targeting sequence, see Table S1 | This paper | N/A |
| HSV for qPCR, see Table S1 | This paper | N/A |
| Recombinant DNA | | |
| pcDNA3.1(+)-his Ub, see Table S1 | This paper | N/A |
| pcDNA3.1(+) | This paper | N/A |
| pGPU6 | This paper | N/A |
| pGPU6-STING shRNA, see Table S1 | This paper | N/A |
| Lenti CRISPR v3 | This paper | N/A |
| v3-STING(WT), see Table S1 | This paper | N/A |
| v3-STING(T229A), see Table S1 | This paper | N/A |
| v3-STING(S4A/S5A), see Table S1 | This paper | N/A |
| v3-STING(S5A), see Table S1 | This paper | N/A |
| v3-STING(S305A), see Table S1 | This paper | N/A |
| v3-STING(S322A), see Table S1 | This paper | N/A |
| v3- STING(S195A), see Table S1 | This paper | N/A |
| v3- STING(T354A), see Table S1 | This paper | N/A |
| v3-STING(T348A/S349A), see Table S1 | This paper | N/A |
| v3- STING(S376A/S379A), see Table S1 | This paper | N/A |
| v3- STING(S379A), see Table S1 | This paper | N/A |
| pMD2.G | This paper | N/A |
| psPAX2 | This paper | N/A |
| **Software and Algorithms** | | |
| Real-time fluorescence quantitative PCR | Roche | Roche Light Cycler480 II |
| ImageJ | NIH | https://imagej.nih.gov/ij |
| Quantity One software | Bio-Rad software | http://softwaretopic.informer.com/quantity-one-software-software/ |
| GraphPad Prism 8 | Graphpad Software | https://www.graphpad.com/ scientiﬁc-software/prism |
| YinOYang 1.2 Server |  | /Services/YinOYang/ |
