# Supplementary figures and images for: O-GlcNAc of STING mediates antiviral innate immunity
Source: Cell Commun Signal. 2024 Mar 1;22:157. doi: 10.1186/s12964-024-01543-8 (PMC10908090; doi:10.1186/s12964-024-01543-8)

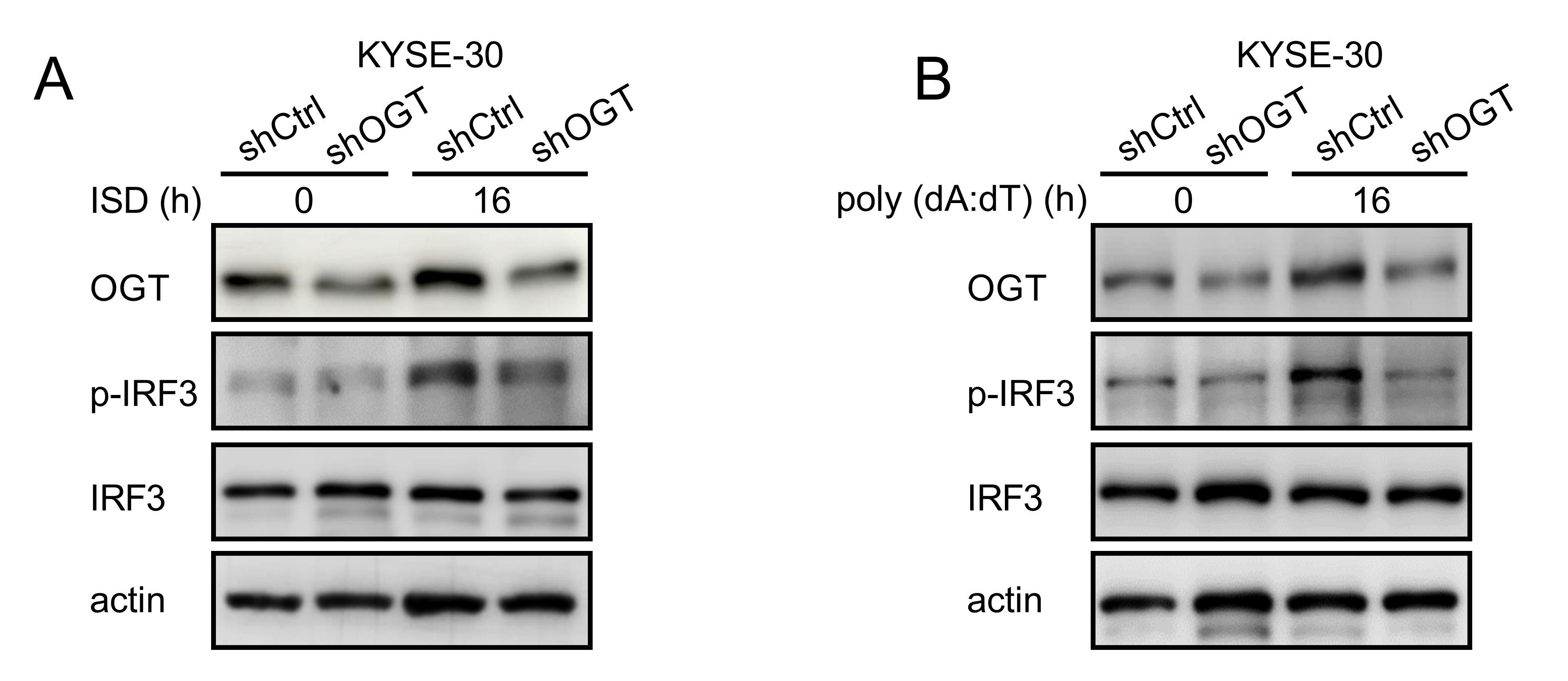

Supplement: Supplementary file 3 — Additional file 3. Figure S1. (A). Immunoblotting of phosphorylated IRF3 (p-IRF3), IRF3, OGT, and actin in shCtrl and shOGT cells infected with ISD (2 μg/mL) for 16 hrs. (B). Immunoblotting of phosphorylated IRF3 (p-IRF3), IRF3, OGT, and actin in shCtrl and shOGT cells infected with poly(dA:dT) (2 μg/mL) for 16 hrs. Data are representatives from 3 independent experiments. Figure S2. (A). O-GlcNAcylated proteins in KYSE-30 cells were pulled down with sWGA beads. cGAS was detected with an anti-cGAS antibody from abcam. (B). Immunoblotting of K63 ubiquitination in immunoprecipitated complex pulled down with STING antibody from lysates of cells treated with or without DON (10 μM) for 12 hrs. STING was immunoprecipitated with anti-STING antibody from abcam. OGT, and STING in the pulldown complex and in the input were detected with immunoblotting. (C). STING was immunoprecipitated with anti-STING antibody from abcam. K63-Ub, OGT, and STING in the pulldown complex and in the input were detected with immunoblotting. (D). HEK-293T cells were transfected with FLAG-tagged STING-WT or -T229A. Co-IP was performed with an anti-STING antibody from abcam. Immunoblotting was performed with antibodies against K27-Ub, K63-Ub, RL2, TRIM56 and STING. Data are representatives from 3 independent experiments. Figure S3. (A). Analyses of STING oligomerization by native gel electrophoresis. cGAMP (9 μg/mL, 16 h) was used to induce high-order oligomerization of STING. The results shown are representatives of three biological repeats. (B). KYSE-30 cells were transfected with FLAG-tagged STING-WT or -T229A and treated with 2 μg/mL ISD for 16 hrs. Cells were then fixed for immunofluorescence detection of STING and calnexin (ER marker) or GM130 (Golgi marker). Data are representatives from 3 independent experiments. Figure S4. KYSE-30 cells reconstituted with either STING-WT or STING-T229E were transfected with 2 μg/mL poly(dA:dT) by Lipofectamine 2000 for 16 hrs. Levels of Ifnb1, Il6, Tnfa, Isg1 [file 12964_2024_1543_MOESM3_ESM.zip › Fig S1.tif]

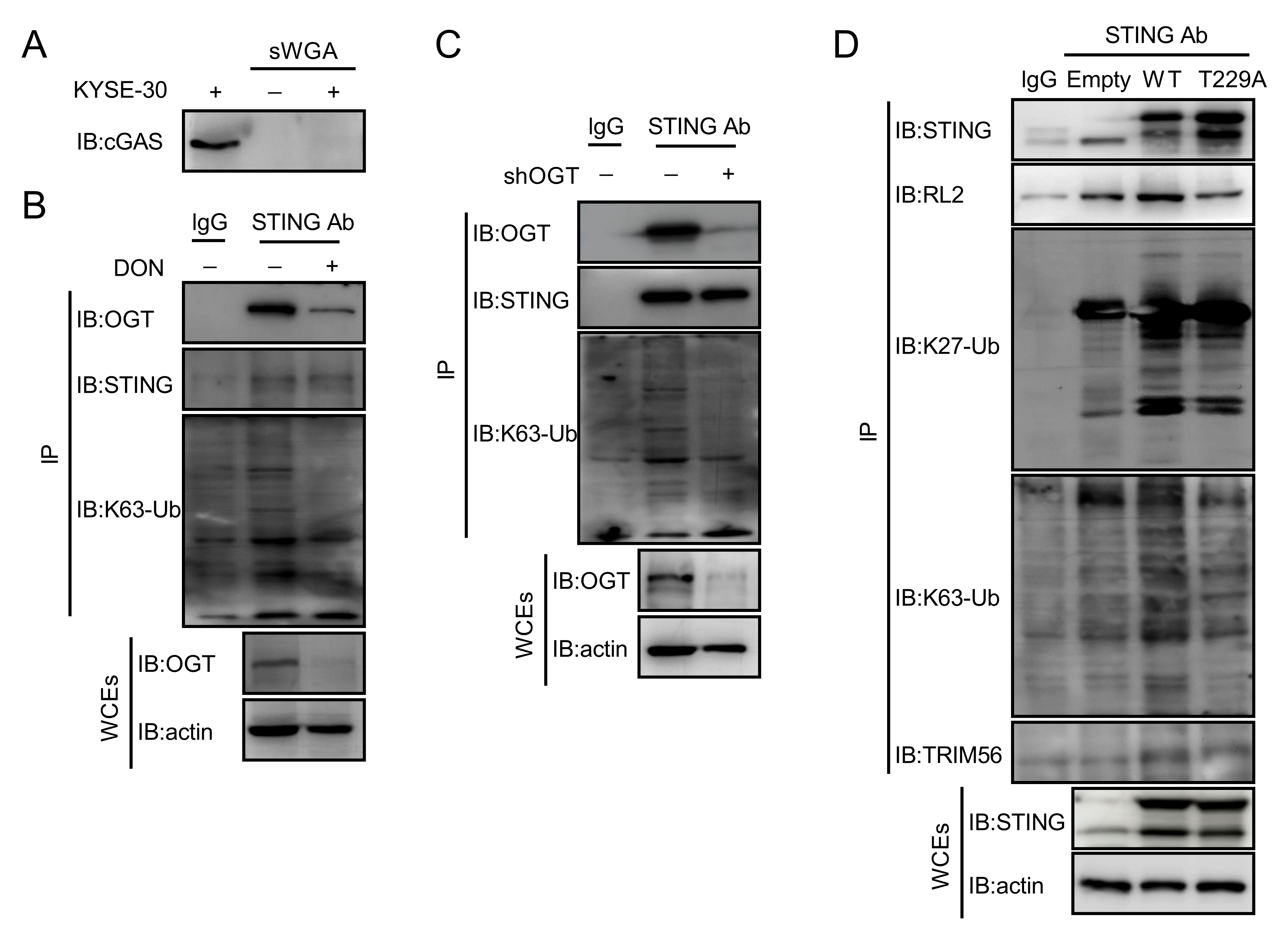

Supplement: Supplementary file 3 — Additional file 3. Figure S1. (A). Immunoblotting of phosphorylated IRF3 (p-IRF3), IRF3, OGT, and actin in shCtrl and shOGT cells infected with ISD (2 μg/mL) for 16 hrs. (B). Immunoblotting of phosphorylated IRF3 (p-IRF3), IRF3, OGT, and actin in shCtrl and shOGT cells infected with poly(dA:dT) (2 μg/mL) for 16 hrs. Data are representatives from 3 independent experiments. Figure S2. (A). O-GlcNAcylated proteins in KYSE-30 cells were pulled down with sWGA beads. cGAS was detected with an anti-cGAS antibody from abcam. (B). Immunoblotting of K63 ubiquitination in immunoprecipitated complex pulled down with STING antibody from lysates of cells treated with or without DON (10 μM) for 12 hrs. STING was immunoprecipitated with anti-STING antibody from abcam. OGT, and STING in the pulldown complex and in the input were detected with immunoblotting. (C). STING was immunoprecipitated with anti-STING antibody from abcam. K63-Ub, OGT, and STING in the pulldown complex and in the input were detected with immunoblotting. (D). HEK-293T cells were transfected with FLAG-tagged STING-WT or -T229A. Co-IP was performed with an anti-STING antibody from abcam. Immunoblotting was performed with antibodies against K27-Ub, K63-Ub, RL2, TRIM56 and STING. Data are representatives from 3 independent experiments. Figure S3. (A). Analyses of STING oligomerization by native gel electrophoresis. cGAMP (9 μg/mL, 16 h) was used to induce high-order oligomerization of STING. The results shown are representatives of three biological repeats. (B). KYSE-30 cells were transfected with FLAG-tagged STING-WT or -T229A and treated with 2 μg/mL ISD for 16 hrs. Cells were then fixed for immunofluorescence detection of STING and calnexin (ER marker) or GM130 (Golgi marker). Data are representatives from 3 independent experiments. Figure S4. KYSE-30 cells reconstituted with either STING-WT or STING-T229E were transfected with 2 μg/mL poly(dA:dT) by Lipofectamine 2000 for 16 hrs. Levels of Ifnb1, Il6, Tnfa, Isg1 [file 12964_2024_1543_MOESM3_ESM.zip › Fig S2.tif]

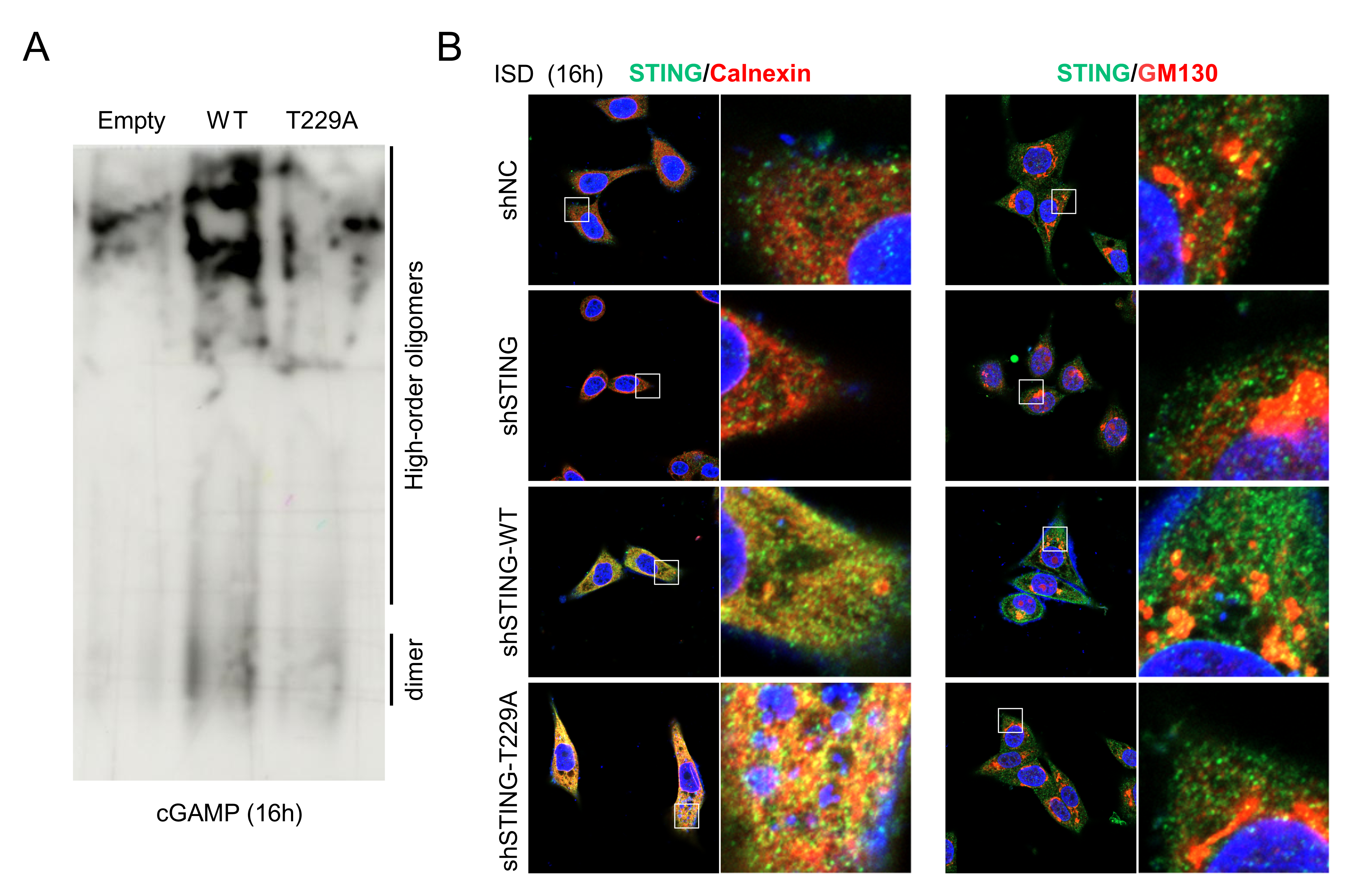

Supplement: Supplementary file 3 — Additional file 3. Figure S1. (A). Immunoblotting of phosphorylated IRF3 (p-IRF3), IRF3, OGT, and actin in shCtrl and shOGT cells infected with ISD (2 μg/mL) for 16 hrs. (B). Immunoblotting of phosphorylated IRF3 (p-IRF3), IRF3, OGT, and actin in shCtrl and shOGT cells infected with poly(dA:dT) (2 μg/mL) for 16 hrs. Data are representatives from 3 independent experiments. Figure S2. (A). O-GlcNAcylated proteins in KYSE-30 cells were pulled down with sWGA beads. cGAS was detected with an anti-cGAS antibody from abcam. (B). Immunoblotting of K63 ubiquitination in immunoprecipitated complex pulled down with STING antibody from lysates of cells treated with or without DON (10 μM) for 12 hrs. STING was immunoprecipitated with anti-STING antibody from abcam. OGT, and STING in the pulldown complex and in the input were detected with immunoblotting. (C). STING was immunoprecipitated with anti-STING antibody from abcam. K63-Ub, OGT, and STING in the pulldown complex and in the input were detected with immunoblotting. (D). HEK-293T cells were transfected with FLAG-tagged STING-WT or -T229A. Co-IP was performed with an anti-STING antibody from abcam. Immunoblotting was performed with antibodies against K27-Ub, K63-Ub, RL2, TRIM56 and STING. Data are representatives from 3 independent experiments. Figure S3. (A). Analyses of STING oligomerization by native gel electrophoresis. cGAMP (9 μg/mL, 16 h) was used to induce high-order oligomerization of STING. The results shown are representatives of three biological repeats. (B). KYSE-30 cells were transfected with FLAG-tagged STING-WT or -T229A and treated with 2 μg/mL ISD for 16 hrs. Cells were then fixed for immunofluorescence detection of STING and calnexin (ER marker) or GM130 (Golgi marker). Data are representatives from 3 independent experiments. Figure S4. KYSE-30 cells reconstituted with either STING-WT or STING-T229E were transfected with 2 μg/mL poly(dA:dT) by Lipofectamine 2000 for 16 hrs. Levels of Ifnb1, Il6, Tnfa, Isg1 [file 12964_2024_1543_MOESM3_ESM.zip › Fig S3.tif]

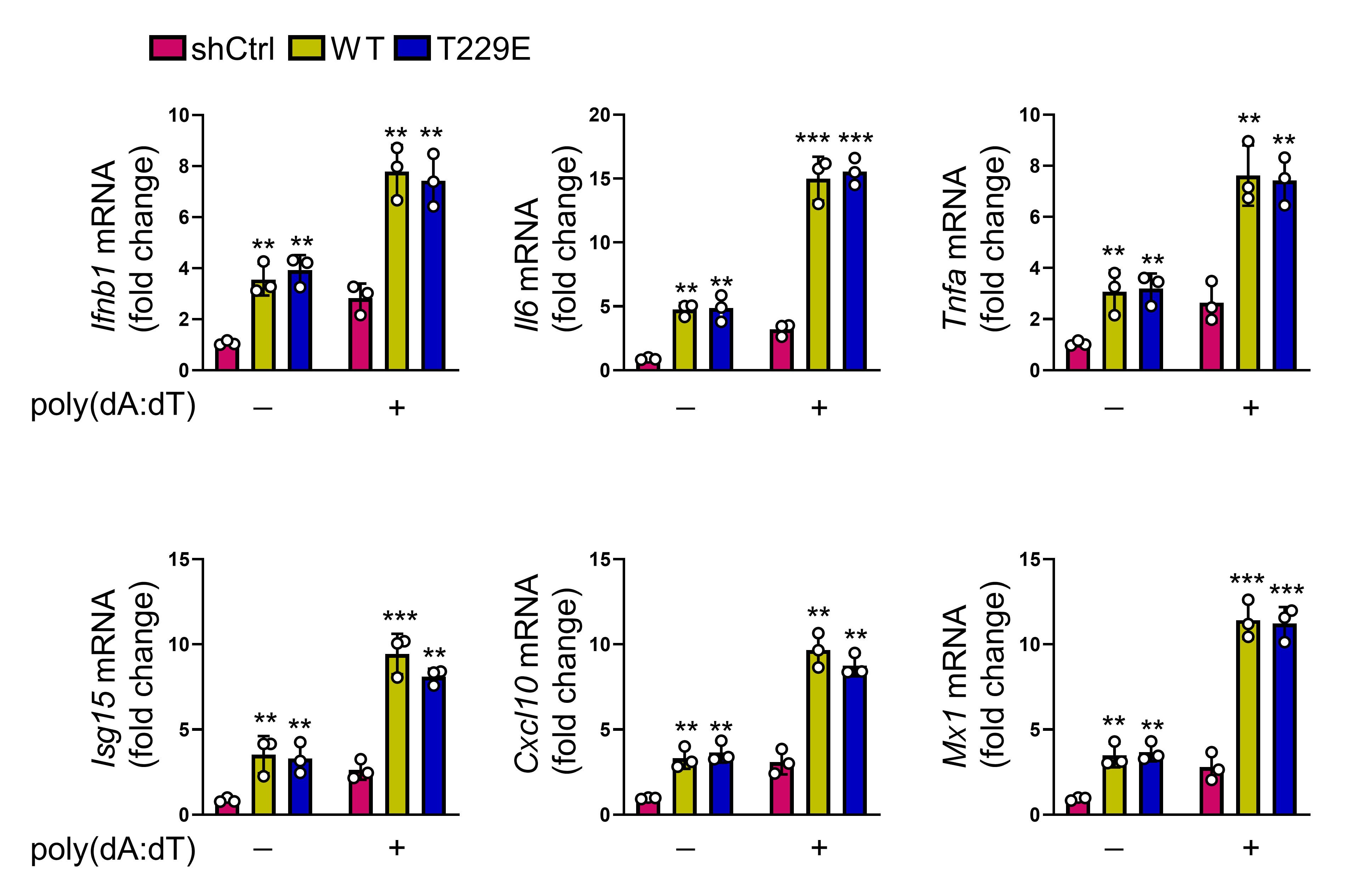

Supplement: Supplementary file 3 — Additional file 3. Figure S1. (A). Immunoblotting of phosphorylated IRF3 (p-IRF3), IRF3, OGT, and actin in shCtrl and shOGT cells infected with ISD (2 μg/mL) for 16 hrs. (B). Immunoblotting of phosphorylated IRF3 (p-IRF3), IRF3, OGT, and actin in shCtrl and shOGT cells infected with poly(dA:dT) (2 μg/mL) for 16 hrs. Data are representatives from 3 independent experiments. Figure S2. (A). O-GlcNAcylated proteins in KYSE-30 cells were pulled down with sWGA beads. cGAS was detected with an anti-cGAS antibody from abcam. (B). Immunoblotting of K63 ubiquitination in immunoprecipitated complex pulled down with STING antibody from lysates of cells treated with or without DON (10 μM) for 12 hrs. STING was immunoprecipitated with anti-STING antibody from abcam. OGT, and STING in the pulldown complex and in the input were detected with immunoblotting. (C). STING was immunoprecipitated with anti-STING antibody from abcam. K63-Ub, OGT, and STING in the pulldown complex and in the input were detected with immunoblotting. (D). HEK-293T cells were transfected with FLAG-tagged STING-WT or -T229A. Co-IP was performed with an anti-STING antibody from abcam. Immunoblotting was performed with antibodies against K27-Ub, K63-Ub, RL2, TRIM56 and STING. Data are representatives from 3 independent experiments. Figure S3. (A). Analyses of STING oligomerization by native gel electrophoresis. cGAMP (9 μg/mL, 16 h) was used to induce high-order oligomerization of STING. The results shown are representatives of three biological repeats. (B). KYSE-30 cells were transfected with FLAG-tagged STING-WT or -T229A and treated with 2 μg/mL ISD for 16 hrs. Cells were then fixed for immunofluorescence detection of STING and calnexin (ER marker) or GM130 (Golgi marker). Data are representatives from 3 independent experiments. Figure S4. KYSE-30 cells reconstituted with either STING-WT or STING-T229E were transfected with 2 μg/mL poly(dA:dT) by Lipofectamine 2000 for 16 hrs. Levels of Ifnb1, Il6, Tnfa, Isg1 [file 12964_2024_1543_MOESM3_ESM.zip › Fig S4.tif]

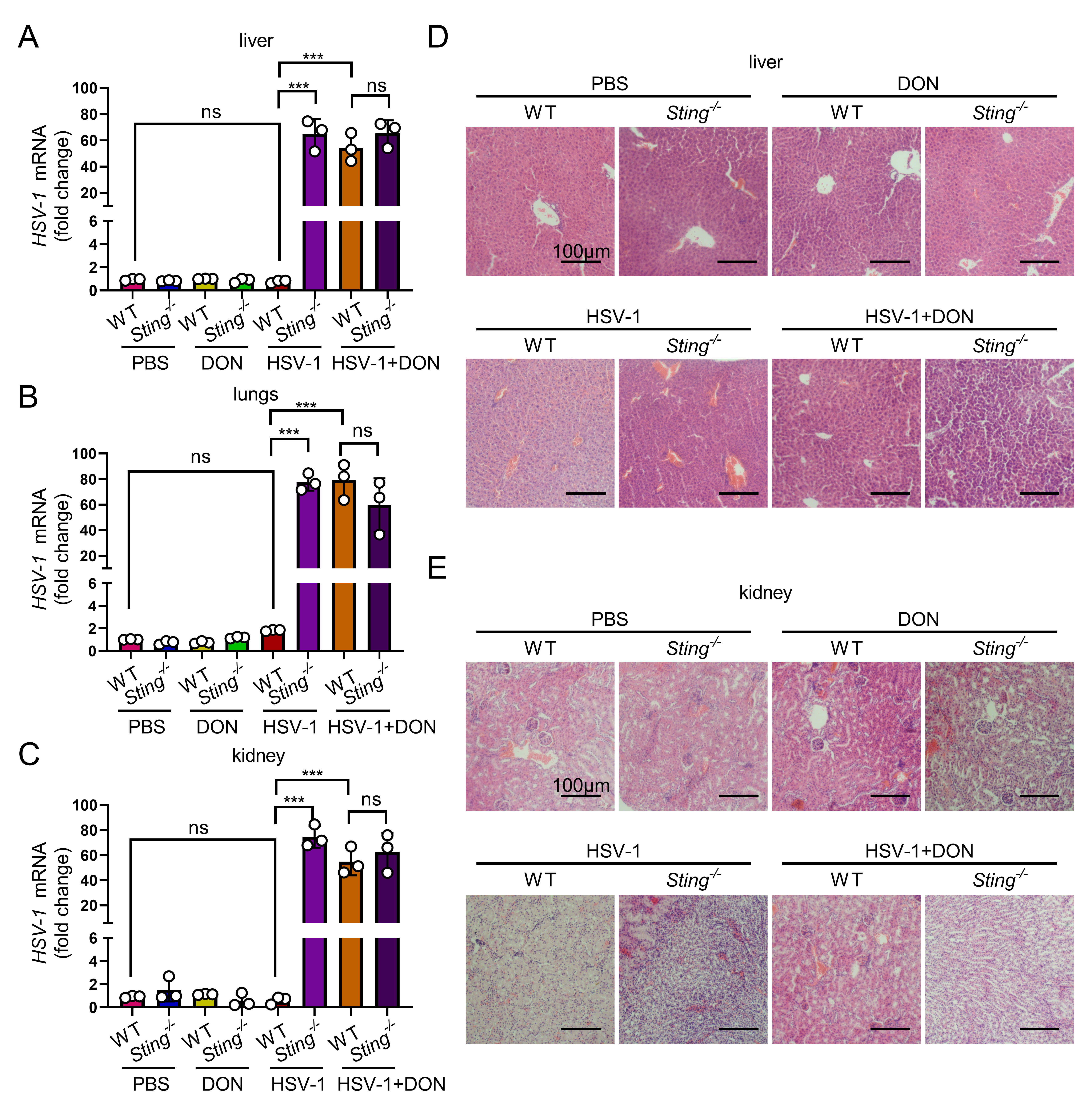

Supplement: Supplementary file 3 — Additional file 3. Figure S1. (A). Immunoblotting of phosphorylated IRF3 (p-IRF3), IRF3, OGT, and actin in shCtrl and shOGT cells infected with ISD (2 μg/mL) for 16 hrs. (B). Immunoblotting of phosphorylated IRF3 (p-IRF3), IRF3, OGT, and actin in shCtrl and shOGT cells infected with poly(dA:dT) (2 μg/mL) for 16 hrs. Data are representatives from 3 independent experiments. Figure S2. (A). O-GlcNAcylated proteins in KYSE-30 cells were pulled down with sWGA beads. cGAS was detected with an anti-cGAS antibody from abcam. (B). Immunoblotting of K63 ubiquitination in immunoprecipitated complex pulled down with STING antibody from lysates of cells treated with or without DON (10 μM) for 12 hrs. STING was immunoprecipitated with anti-STING antibody from abcam. OGT, and STING in the pulldown complex and in the input were detected with immunoblotting. (C). STING was immunoprecipitated with anti-STING antibody from abcam. K63-Ub, OGT, and STING in the pulldown complex and in the input were detected with immunoblotting. (D). HEK-293T cells were transfected with FLAG-tagged STING-WT or -T229A. Co-IP was performed with an anti-STING antibody from abcam. Immunoblotting was performed with antibodies against K27-Ub, K63-Ub, RL2, TRIM56 and STING. Data are representatives from 3 independent experiments. Figure S3. (A). Analyses of STING oligomerization by native gel electrophoresis. cGAMP (9 μg/mL, 16 h) was used to induce high-order oligomerization of STING. The results shown are representatives of three biological repeats. (B). KYSE-30 cells were transfected with FLAG-tagged STING-WT or -T229A and treated with 2 μg/mL ISD for 16 hrs. Cells were then fixed for immunofluorescence detection of STING and calnexin (ER marker) or GM130 (Golgi marker). Data are representatives from 3 independent experiments. Figure S4. KYSE-30 cells reconstituted with either STING-WT or STING-T229E were transfected with 2 μg/mL poly(dA:dT) by Lipofectamine 2000 for 16 hrs. Levels of Ifnb1, Il6, Tnfa, Isg1 [file 12964_2024_1543_MOESM3_ESM.zip › Fig S5.tif]

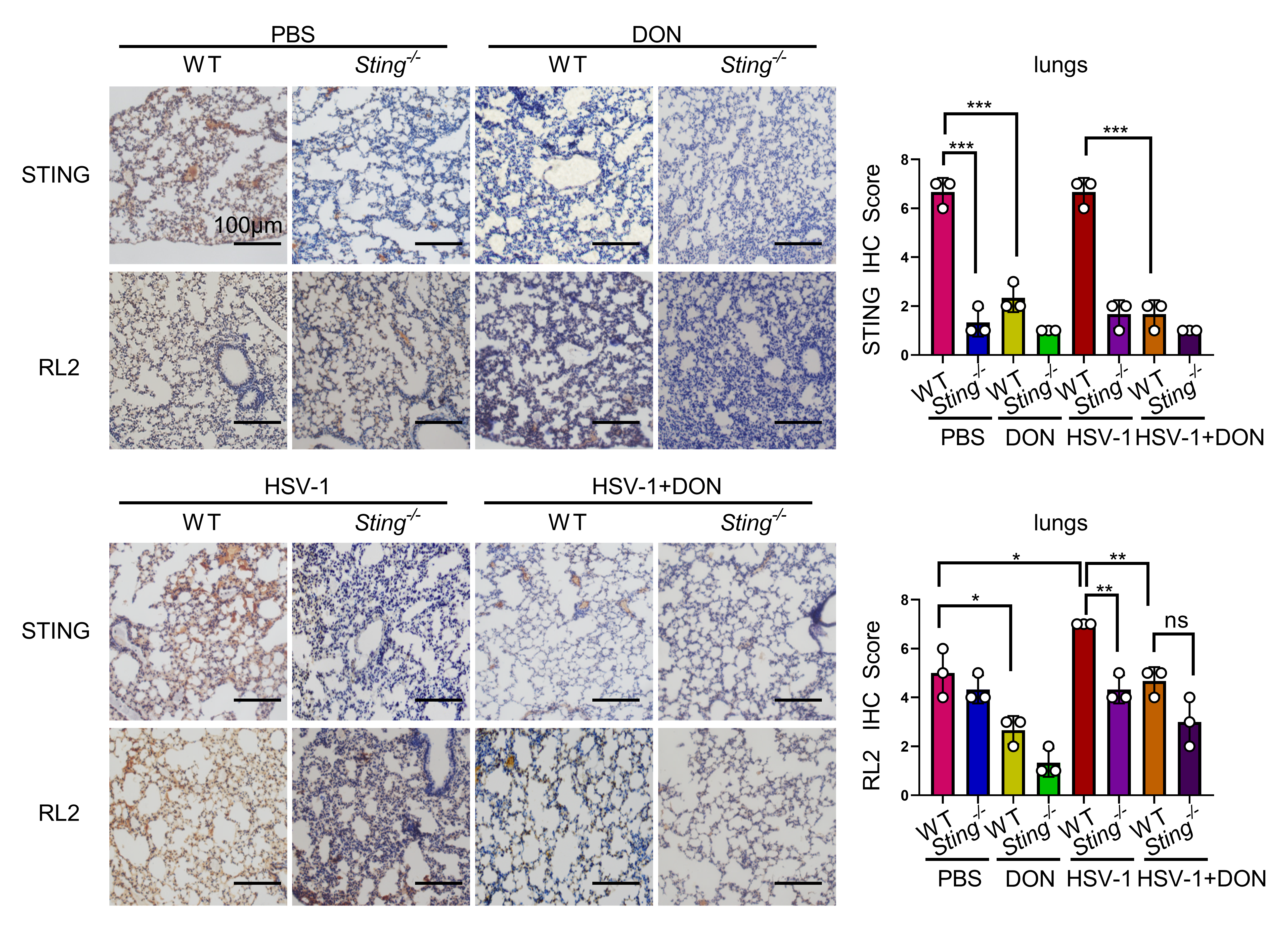

Supplement: Supplementary file 3 — Additional file 3. Figure S1. (A). Immunoblotting of phosphorylated IRF3 (p-IRF3), IRF3, OGT, and actin in shCtrl and shOGT cells infected with ISD (2 μg/mL) for 16 hrs. (B). Immunoblotting of phosphorylated IRF3 (p-IRF3), IRF3, OGT, and actin in shCtrl and shOGT cells infected with poly(dA:dT) (2 μg/mL) for 16 hrs. Data are representatives from 3 independent experiments. Figure S2. (A). O-GlcNAcylated proteins in KYSE-30 cells were pulled down with sWGA beads. cGAS was detected with an anti-cGAS antibody from abcam. (B). Immunoblotting of K63 ubiquitination in immunoprecipitated complex pulled down with STING antibody from lysates of cells treated with or without DON (10 μM) for 12 hrs. STING was immunoprecipitated with anti-STING antibody from abcam. OGT, and STING in the pulldown complex and in the input were detected with immunoblotting. (C). STING was immunoprecipitated with anti-STING antibody from abcam. K63-Ub, OGT, and STING in the pulldown complex and in the input were detected with immunoblotting. (D). HEK-293T cells were transfected with FLAG-tagged STING-WT or -T229A. Co-IP was performed with an anti-STING antibody from abcam. Immunoblotting was performed with antibodies against K27-Ub, K63-Ub, RL2, TRIM56 and STING. Data are representatives from 3 independent experiments. Figure S3. (A). Analyses of STING oligomerization by native gel electrophoresis. cGAMP (9 μg/mL, 16 h) was used to induce high-order oligomerization of STING. The results shown are representatives of three biological repeats. (B). KYSE-30 cells were transfected with FLAG-tagged STING-WT or -T229A and treated with 2 μg/mL ISD for 16 hrs. Cells were then fixed for immunofluorescence detection of STING and calnexin (ER marker) or GM130 (Golgi marker). Data are representatives from 3 independent experiments. Figure S4. KYSE-30 cells reconstituted with either STING-WT or STING-T229E were transfected with 2 μg/mL poly(dA:dT) by Lipofectamine 2000 for 16 hrs. Levels of Ifnb1, Il6, Tnfa, Isg1 [file 12964_2024_1543_MOESM3_ESM.zip › Fig S6.tif]

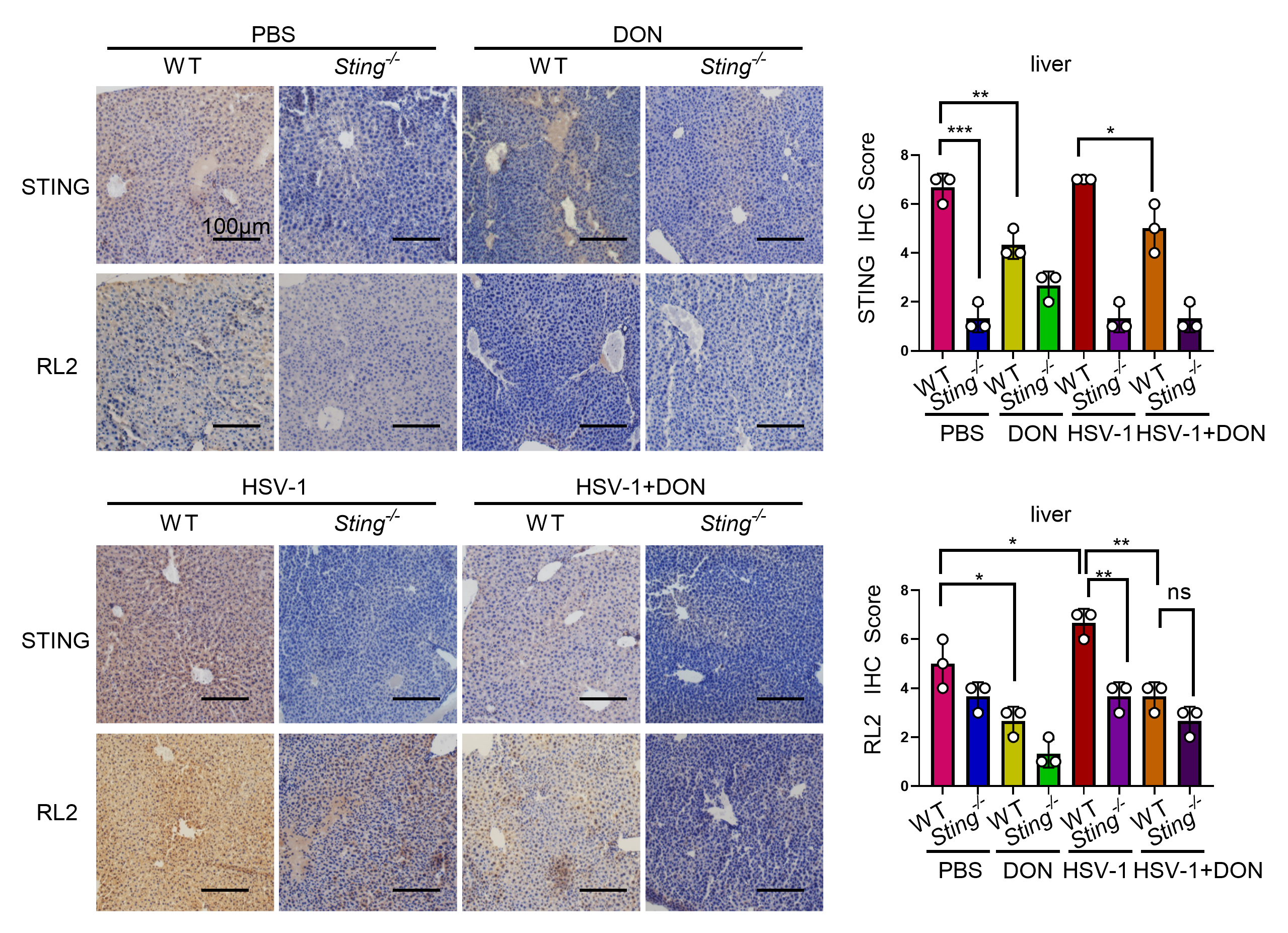

Supplement: Supplementary file 3 — Additional file 3. Figure S1. (A). Immunoblotting of phosphorylated IRF3 (p-IRF3), IRF3, OGT, and actin in shCtrl and shOGT cells infected with ISD (2 μg/mL) for 16 hrs. (B). Immunoblotting of phosphorylated IRF3 (p-IRF3), IRF3, OGT, and actin in shCtrl and shOGT cells infected with poly(dA:dT) (2 μg/mL) for 16 hrs. Data are representatives from 3 independent experiments. Figure S2. (A). O-GlcNAcylated proteins in KYSE-30 cells were pulled down with sWGA beads. cGAS was detected with an anti-cGAS antibody from abcam. (B). Immunoblotting of K63 ubiquitination in immunoprecipitated complex pulled down with STING antibody from lysates of cells treated with or without DON (10 μM) for 12 hrs. STING was immunoprecipitated with anti-STING antibody from abcam. OGT, and STING in the pulldown complex and in the input were detected with immunoblotting. (C). STING was immunoprecipitated with anti-STING antibody from abcam. K63-Ub, OGT, and STING in the pulldown complex and in the input were detected with immunoblotting. (D). HEK-293T cells were transfected with FLAG-tagged STING-WT or -T229A. Co-IP was performed with an anti-STING antibody from abcam. Immunoblotting was performed with antibodies against K27-Ub, K63-Ub, RL2, TRIM56 and STING. Data are representatives from 3 independent experiments. Figure S3. (A). Analyses of STING oligomerization by native gel electrophoresis. cGAMP (9 μg/mL, 16 h) was used to induce high-order oligomerization of STING. The results shown are representatives of three biological repeats. (B). KYSE-30 cells were transfected with FLAG-tagged STING-WT or -T229A and treated with 2 μg/mL ISD for 16 hrs. Cells were then fixed for immunofluorescence detection of STING and calnexin (ER marker) or GM130 (Golgi marker). Data are representatives from 3 independent experiments. Figure S4. KYSE-30 cells reconstituted with either STING-WT or STING-T229E were transfected with 2 μg/mL poly(dA:dT) by Lipofectamine 2000 for 16 hrs. Levels of Ifnb1, Il6, Tnfa, Isg1 [file 12964_2024_1543_MOESM3_ESM.zip › Fig S7.tif]
